# Stimuli-responsive nanodrug self-assembled from amphiphilic drug-inhibitor conjugate for overcoming multidrug resistance in cancer treatment

**DOI:** 10.7150/thno.36163

**Published:** 2019-08-12

**Authors:** Ping Huang, Guanchun Wang, Yue Su, Yongfeng Zhou, Wei Huang, Rong Zhang, Deyue Yan

**Affiliations:** 1 School of Chemistry and Chemical Engineering, State Key Laboratory of Metal Matrix Composites, Shanghai Jiao Tong University, 800 Dongchuan Road, Shanghai 200240, P. R. China; 2 Department of Obstetrics and Gynecology, Fengxian Hospital, Southern Medical University, Shanghai 201499, China

**Keywords:** multidrug resistance, stimuli-responsive, drug-inhibitor conjugate, self-assembly, nanodrug

## Abstract

Severe multidrug resistance (MDR) often develops in the process of chemotherapy for most small molecule anticancer drugs, which results in clinical chemotherapy failures.

**Methods:** Here, a nanodrug is constructed through the self-assembly of amphiphilic drug-inhibitor conjugates (ADIC) containing a redox-responsive linkage for reversing the multidrug resistance (MDR) in cancer treatment. Specifically, hydrophilic anticancer irinotecan (Ir) and hydrophobic P-gp protein inhibitor quinine (Qu) are linked by a redox responsive bridge for overcoming MDR of tumors.

**Results:** Ir-ss-Qu is able to self-assemble into nanoparticles (NPs) in water and shows the longer blood retention half-life compared with that of free Ir or Qu, which facilitates drug accumulation in tumor site. After endocytosis of Ir-ss-Qu NPs by drug-resistant tumor cells, the disulfide bond in the linkage between Ir and Qu is cleaved rapidly induced by glutathione (GSH) to release anticancer drug Ir and inhibitor Qu synchronously. The released Qu can markedly reduce the expression of P-gp in drug-resistant tumor cells and inhibits P-gp to pump Ir out of the cells. The increased concentration of intracellular Ir can effectively improve the therapeutic efficacy.

**Conclusions:** Such redox-responsive Ir-ss-Qu NPs, as a drug delivery system, exhibit very high cytotoxicity and the most effective inhibitory to the growth of drug-resistant breast cancer compared with that of free therapeutic agents *in vitro* and* in vivo*.

## Introduction

Nowadays chemotherapy is still one of most primary approaches for cancer treatments in clinic. However, multidrug resistance (MDR) that happens frequently in chemotherapy has become a serious problem for many cancer patients. Among numerous complicated mechanisms of MDR, the over- expression of drug efflux pumps, such as P-glycoprotein (P-gp), belonging to the family of ATP-binding cassette (ABC) transporter, is one of the most major cause 1. Overexpression of P-gp is capable of extruding various small molecular anticancer drugs, such as doxorubicin, paclitaxel, cisplatin etc., out of the cancer cells to decrease their intracellular accumulation dramatically, which results in the lower therapeutic efficacy 2-4. Therefore, the inhibition of P-gp expression has become one of most effective approaches to reverse MDR and enhance the efficacy of chemotherapy 5. At present, it is a popular way in clinical cancer therapy to overcome MDR by using a simple physical mixture of a chemotherapeutic agent and a P-gp inhibitor. However, most of results are not satisfied due to the inherent drawbacks of P-gp inhibitor, including poor bioavailability, rapid blood/renal clearance, low selectivity, inherent toxicity, and pharmacokinetic difference with anticancer drugs 6,7. To address above dilemmas, nanoparticles-based drug delivery systems (DDSs), including polymer nanoparticles 8-13, liposomes 14,15, metal-organic frameworks (MOFs) 16, inorganic nanoparticles 17-20, have been extensively proposed to reverse MDR since they can increase bioavailability and accumulation of the drug and inhibitor at tumor sites through the enhanced permeation and retention (EPR) effect 21,22 .

**Figure 1:**
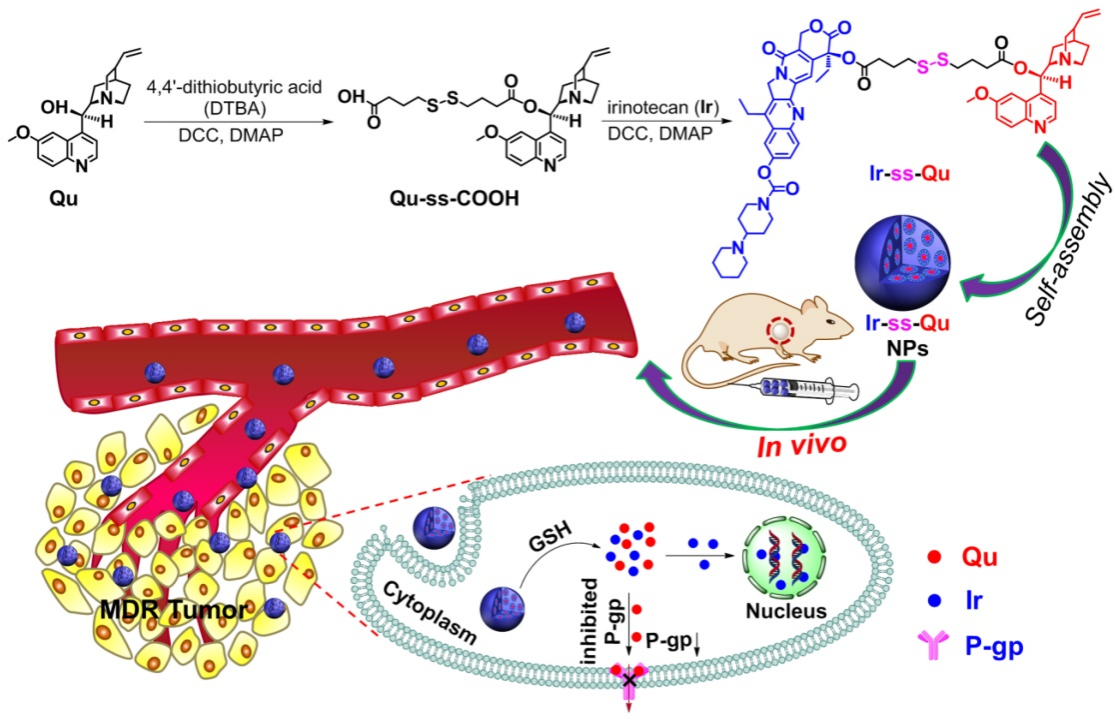
Synthetic route of Ir-ss-Qu conjugate and schematic of the self-assembled Ir-ss-Qu conjugate NPs for reversing MDR of tumor *in vivo*.

As an optimal drug delivery system for overcoming MDR, the drug and the inhibitor should be rapidly and thoroughly released into cytoplasm, which leads to a lower expression of P-gp while a sufficiently high intracellular drug concentration in drug-resistant cancer cells and then kill the cells effectively 23. Therefore, a large number of stimuli-responsive nanocarriers for various tumor microenvironments (e.g., pH 24,25, redox 26-32, enzymes 33, and ROS 34) have been developed and used as DDSs for overcoming MDR in last two decades 35,36. Especially redox-responsive nanocarriers containing disulfide linkages for overcoming MDR have attracted more attention because they can degrade rapidly and specifically in respond to the redox microenvironment in cancer cells, where the concentration of glutathione (GSH) is one order of magnitude higher than that in normal cells and blood environment 37,38. Unfortunately such promising stimuli-responsive nanocarriers for reversing MDR still suffer from several limitations, including low drug loading capacity, undefined molecular weight and structure, no therapeutic efficacy. In addition, most of them often cause serious toxicity and inflammation to kidneys and other organs in the course of degradation, metabolism, and excretion. To solve above problems, it is necessary to construct a stimuli-responsive drug delivery system without any carriers, which simply consists of anti-cancer drug, P-gp inhibitor and a linkage group for overcoming MDR. Herein we synthesized an amphiphilic small molecule anticancer drug-inhibitor conjugate to construct nanodrug for overcoming MDR. In detail, the conjugate (Ir-ss-Qu) is composed of a hydrophilic anticancer drug Ir, a hydrophobic inhibitor Qu and a redox-responsive linkage. Qu servers as a P-gp protein inhibitor 39,40, which can down-regulate the expression of P-gp in drug-resistant cells. Ir is a water-soluble anti-cancer derivative of camptothecin, which can induce the apoptosis of cancer cells by inhibiting DNA topoisomerase I. Owing to its amphiphilic structure, Ir-ss-Qu conjugate can self-assemble into NPs (Ir-ss-Qu NPs) in water. After Ir-ss-Qu NPs internalized by drug-resistant cancer cells, Ir and Qu are released synchronously by GSH-triggered cleavage of disulfide bond in the linkage. The released Qu could down-regulate the expression of P-gp effectively and prevent the released anticancer drug Ir being pumped out, which markedly increases the concentration of Ir in drug-resistant cells and finally kill the cells (Figure 1).

## Results and Discussion

### Synthesis and self-assembly of redox-responsive Ir-ss-Qu conjugate

**Figure 2:**
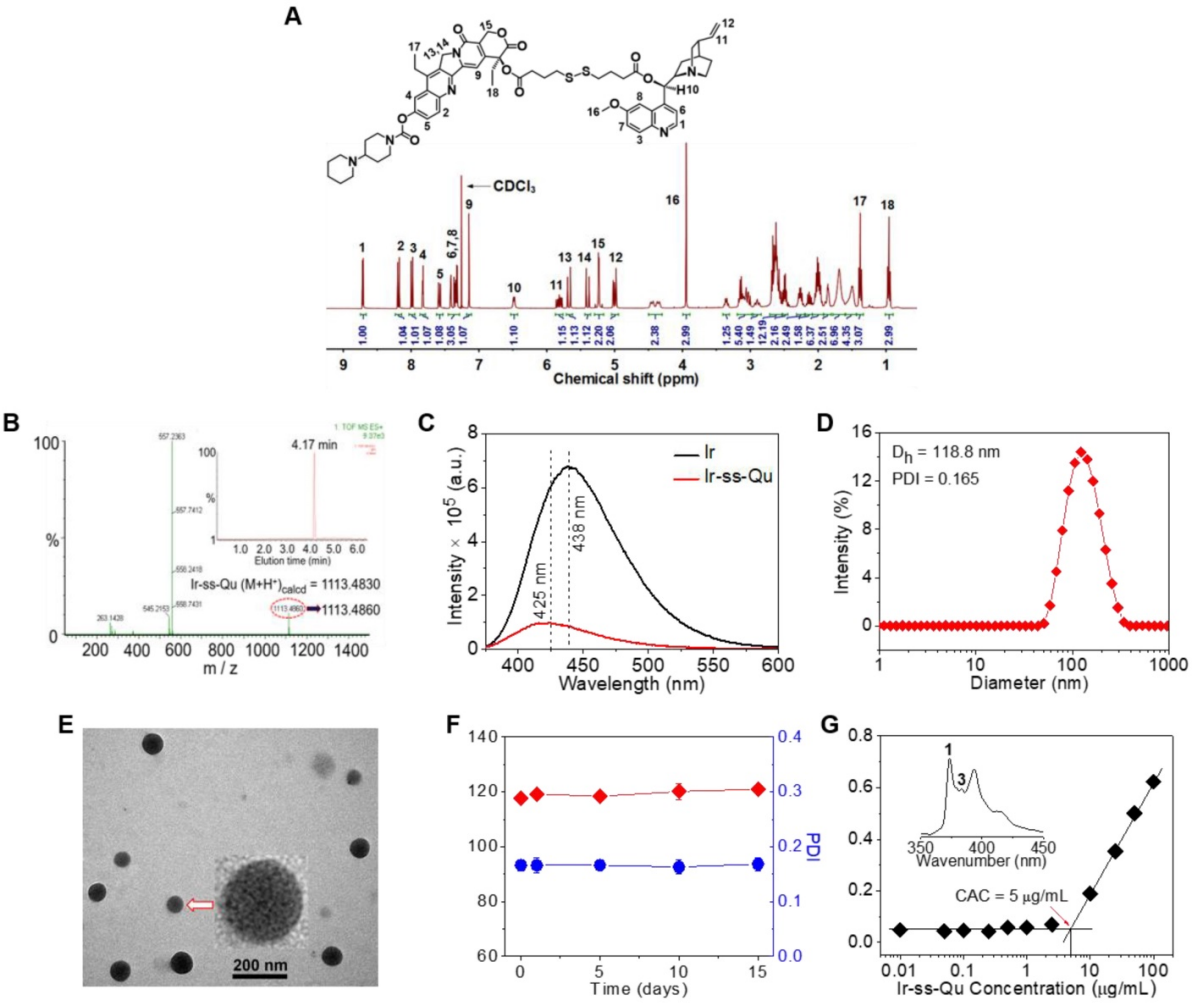
Chemical characterization of Ir-ss-Qu conjugate and its self-assembled NPs. (**A**) ^1^H NMR spectrum of Ir-ss-Qu conjugate in CDCl_3_. (**B**) Mass spectrum of Ir-ss-Qu (ESI-MS m/z (M+H^+^) calcd 1113.4830, found 1113.4860 (M+H^+^). Inset: LC profile of Ir-ss-Qu. Retention time (tR) is 4.17 min. (**C**) Fluorescence emission spectra of Ir (λ_ex_ = 360 nm, λ_em_ = 438 nm) and Ir-ss-Qu conjugate (λ_ex_ = 360 nm, λ_em_ = 425 nm) in water at concentration of 0.5 μM. (**D**) DLS curve of Ir-ss-Qu conjugate NPs shows the diameter distribution of the NPs, the average size (D_h_ = 118.8 nm) and the polydispersity index (PDI = 0.165). (**E**) TEM image of Ir-ss-Qu NPs. Inset: the enlarged TEM image of one nanoparticle. Scale bars: 200 nm. (**F**) Influence of storage on diameter (red) and PDI (blue) of Ir-ss-Qu NPs. The solution of Ir-ss-Qu NPs was stored at 4 °C in refrigerator for 15 days. At different time intervals (0, 1, 5, 10, and 15 d), the average size and PDI were determined. Samples were measured in triplicates. The values are the mean ± SD. (**G**) Relationship between the fluorescent intensity ratio (*I*_3_/*I*_1_) and Ir-ss-Qu conjugate concentration in water. Inset: the fluorescence emission spectrum of pyrene in aqueous solution. The CAC value is about 5 μg mL^-1^.

Due to Ir and Qu containing a reactive hydroxyl group respectively, 4,4'-dithiobutyric acid (DTBA) was selected as a linkage to couple both of them to form a redox-responsive amphiphilic conjugate (Ir-ss- Qu). The synthetic process of Ir-ss-Qu via two-step esterification was exhibited in Figure 1. First, Qu was reacted with excess DTBA to yield an intermediate product (Qu-ss-COOH). The chemical structure of Qu-ss-COOH was verified by ^1^H NMR, ^13^C NMR and LC-MS analyses (Figure S1, Figure S2). Then Ir-ss-Qu conjugate was obtained via the esterification of Ir and Qu-ss-COOH at room temperature. The ^1^H NMR and ^13^C NMR spectra of Ir-ss-Qu conjugate were shown in Figure 2A and Figure S3. In Figure 2A, the integral ratio of all proton signals of *I*_1_:*I*_2_:*I*_3_:*I*_4_:*I*_5_:*I*_6_:*I*_7_:*I*_8_:*I*_9_:*I*_10_:*I*_11_: *I*_12_:*I*_13_:*I*_14_:*I*_15_:*I*_16_:*I*_17_:*I*_18_ approximately equals to 1:1:1:1:1: 1:1:1:1:1:1:2:1:1:2:3:3:3, which is in accordance with the proton ratio of Ir-ss-Qu conjugate. Except for the solvent signal of chloroform, no other signals of impurities were observed. The ^13^C NMR spectrum also confirmed the chemical structure of Ir-ss-Qu conjugate. The LC-MS data of Ir-ss-Qu conjugate was shown in Figure 2B. The measured molecular weight of Ir-ss-Qu conjugate (m/z, M+H^+^) was 1113.4860, which was in agreement with the calculated value (m/z, M+H^+^, 1113.4830). Only one retention time of Ir-ss-Qu conjugate was found at 4.17 min in its LC profile in the inset of Figure 2B, which indicated the high purity of Ir-ss-Qu conjugate. Moreover, Ir-ss-Qu conjugate was also characterized by ultraviolet-visible spectrophotometer (UV-vis) and Fourier transform infrared spectroscopy (FTIR) and the results displayed in Figure S4, Figure S5. In addition, the fluorescence of Ir and Ir-ss-Qu conjugate was further examined and the results were shown in Figure 2C. Ir itself can emit strong blue fluorescence under the irradiation of UV-lamp and the maximum emission wavelength was at 438 nm. After conjugating Qu onto Ir through the linkage of DTBA containing disulfide bond, the maximum emission wavelength of Ir-ss-Qu conjugate shifted from 438 nm to 425 nm and the fluorescence intensity was decreased 7-fold. We also investigated the fluorescence behavior of Ir-ss-Qu conjugates treated with GSH (5 mM, the intracellular concentration) for different time. The fluorescence emission intensity (λ_ex_ = 360 nm, λ_em_ = 425 nm) of Ir-ss-Qu conjugates increased 5-fold when they were treated with GSH for 12 h, which was attributed to the disulfide bond in Ir-ss-Qu conjugate cleaved under the reduction condition to release Ir (Figure S6). Meanwhile, the fluorescence emission intensity of free Ir (0.5 μM) was not changed when it was treated with GSH (5 mM) for a similar period of time (Figure S7). This indicated that Qu was able to quench the fluorescence of Ir when it was conjugated with Ir. On the contrary, the fluorescence of Ir was recovered again when the disulfide bond in Ir-ss-Qu conjugate was cleaved triggered by GSH. All above results confirmed that Ir-ss-Qu conjugate was synthesized successfully.

Owing to its amphiphilic molecular structure, Ir-ss-Qu conjugate could self-assemble into NPs in water. The Ir-ss-Qu NPs were obtained according to the following process: the dimethyl sulfoxide (DMSO) solution of Ir-ss-Qu conjugate was added dropwise into water and then removed DMSO by dialysis against water. The size and morphology of the resulting NPs were determined by dynamic light scattering (DLS) and transmission electron microscopy (TEM) measurements. The DLS curve in Figure 2D showed the average size of Ir-ss-Qu NPs was approximately 118.8 nm with a narrow unimodal distribution of 0.165. The surface charge of Ir-ss-Qu NPs in PBS solution (pH 7.4) was also measured by DLS and the zeta-potential of them was + 5.8 mV, which indicated the positive charges were on the surface of Ir-ss-Qu NPs. The TEM image of Ir-ss-Qu NPs in Figure 2E exhibited the spherical aggregates with an average size of approximately 102 nm, which was slightly smaller than that measured by DLS, which was attributed to the shrinkage of aggregates in a drying situation of TEM sample. A representative enlarged TEM image of one Ir-ss-Qu nanoparticle was displayed in the inset of Figure 2E. It clearly exhibited that the Ir-ss-Qu NP was composed of a lot of small black domains surrounded by some light grey continuous hydrophilic phase, which indicated that its self-assembly also followed the mechanism of our previous report 41. The storage stability of Ir-ss-Qu NPs was investigated by monitoring their average hydrodynamic diameter and the polydispersity index (PDI) for different time intervals. The results were revealed in Figure 2F and no significant change was found in diameter and PDI of Ir-ss-Qu NPs, which indicated they were extremely stable stored at 4 °C in refrigerator for 15 days.

The critical aggregation concentration (CAC) of Ir-ss-Qu conjugate in water was determined by the method of pyrene as a fluorescent probe. In the fluorescence spectrum of pyrene, the emission intensity of first and third bands was labeled as *I*_1_ and *I*_3_, respectively (the inset of Figure 2G). The ratio of *I*_3_/*I*_1_ was closely related to the polarity of the medium surrounding pyrene molecules 42. The relationship of the *I*_3_/*I*_1_ ratio with the concentration of Ir-ss-Qu conjugate was exhibited in Figure 2G. The value of *I*_3_/*I*_1_ was nearly unchanged at the low concentration of Ir-ss-Qu conjugate, whereas the value began to increase dramatically with the concentration of Ir-ss-Qu conjugate increasing, which suggested the pyrene in a hydrophobic environment at a certain concentration of Ir-ss-Qu conjugate. The CAC value of Ir-ss-Qu conjugate was about 5 μg mL^-1^ on the base of the inflection point of the curve.

### Redox-triggered disassembly of Ir-ss-Qu NPs and drug release

**Figure 3:**
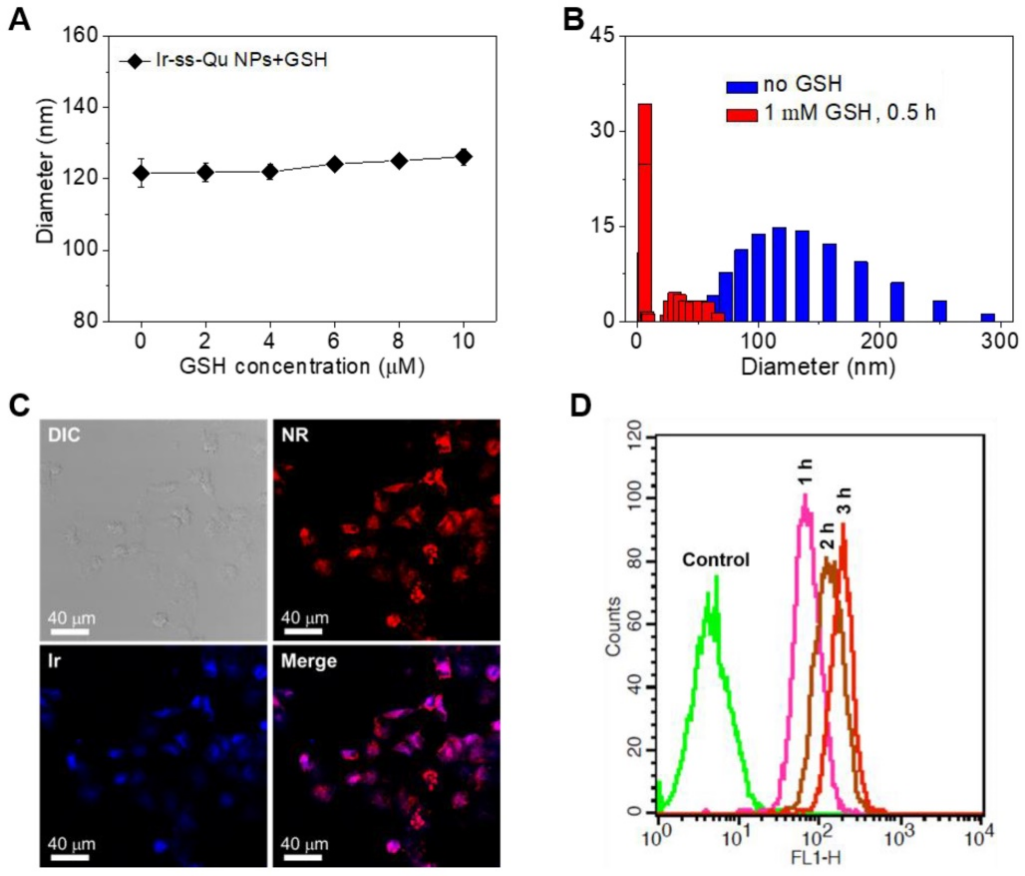
(**A**) The diameter of Ir-ss-Qu NPs treated with different GSH concentrations (2, 4, 6, and 10 μM, blood's GSH concentration) for 1 h, the average size were determined. Samples were measured in triplicates. The values are the mean ± SD. (**B**) The size change of Ir-ss-Qu conjugate in response to 1 mM GSH (intracellular concentration) determined by DLS measurement. (**C**) CLSM photos of MCF-7/ADR cells incubated with NR-loaded Ir-ss-Qu conjugate NPs for 3 h. (**D**) Representative flow cytometry histogram profiles of MCF-7/ADR cells cultured with NR-loaded Ir-ss-Qu conjugate NPs for 3 h, the untreated cells are used as a control.

For human body, generally the concentration of GSH was micromole level (~10 μM) in the blood plasma and extracellular environment, whereas millimole level (~10 mM) in the cytoplasm 43-45. According to previous report, disulfide bonds were stable under physiological environment in blood circulation and extracellular conditions due to the relatively lower concentration of GSH, but cleaved rapidly in cancer cells due to the relatively high reductive environment 46. Therefore, we first investigated the stability of Ir-ss-Qu NPs under the micromole concentration of GSH in blood circulation. The diameter of Ir-ss-Qu NPs was measured by DLS after they were incubated with GSH at the different concentration from 2 μM to 10 μM (micromole level) for 1 h. As displayed in Figure 3A, only a nominal diameter increase was observed, which indicated the high stability of Ir-ss-Qu NPs in the bloodstream. Meanwhile, the time-dependent diameter change of Ir-ss-Qu NPs treated with 10 μM GSH and 10% FBS was also monitored by DLS and the results were shown in Figure S8, which further confirmed the high stability of Ir-ss-Qu NPs under the physiological condition or the serum. When the concentration of GSH was increased to the millimole level, the diameter change of Ir-ss-Qu NPs was also studied by DLS measurement and the results were shown in Figure 3B. Notably, the average diameter of Ir-ss-Qu NPs dramatically decreased from ~120 nm to ~25 nm with a broad bimodal distribution after they were treated with 1 mM GSH for only 0.5 h. The morphology of the disassembly was observed by TEM and the results were shown in Figure S9. No spherical nanoparticles were observed and only some irregular fragments appeared in the TEM image. The disassembly of Ir-ss-Qu NPs was attributed to the cleavage of disulfide bonds under the millimole level of GSH, which was similar to the intracellular concentration of GSH. The Ir release behavior of Ir-ss-Qu NPs *in vitro* was evaluated by dialysis in PBS (pH 7.4) at 37 °C without or with the treatment of GSH (1 mM). The Ir release curve in Figure S10 indicated that the total Ir release amount of Ir-ss-Qu NPs was relatively low (~15%) without the treatment of GSH for 12 h. On the contrary, with the treatment of GSH (1 mM), the Ir release of Ir-ss-Qu NPs was accelerated significantly and the total Ir release amount was up to about 60% for 12 h.

The previous report demonstrated that the conjugated prodrug coupled with the linkage of DTBA containing disulfide bond could be reverted to the original drugs under the reductive conditions 47. Accordingly, we speculated that Ir-ss-Qu conjugates would be converted into free Ir and Qu on the base of the reported reductive mechanism (Figure S11). In order to verify above speculation, LC-MS technique was adopted to monitor the change of Ir-ss-Qu conjugate treated with GSH. As shown in Figure S12A, a new signal of the retention time appeared at 4.50 min after GSH was added into the aqueous solution of Ir-ss-Qu conjugate for 15 min, which indicated that the disulfide bond in Ir-ss-Qu conjugate reacted with GSH to form a new product. With the treatment time increased to 4 h, another signal of the retention time appeared at 4.95 min except for the signal at 4.50 min and simultaneously the signal intensity was significantly improved, which indicated that the cleavage of disulfide bond in Ir-ss-Qu conjugate was accelerated obviously under the treatment of GSH. Analyzed by the extracted ion chromatography assay, the resulting product was composed of Ir-SH, Qu-SH, free Ir, and Qu (Figure S12B-I), which was in accord with the reported reductive mechanism. In other words, Ir-ss-Qu conjugates were able to recover to free Ir and Qu eventually under the reductive condition.

Here Nile red (NR) was used as a fluorescent probe and loaded into Ir-ss-Qu conjugate NPs. Then the NR-loaded Ir-ss- Qu NPs were adopted to study their ability to enter into drug- resistant cancer cells and rapidly release the drug under the intracellular reductive condition in MDR cancer cells. The DLS and TEM measurement results of NR-loaded Ir-ss-Qu NPs were shown in Figure S13. Both average size and polydispersity index of NR-loaded Ir-ss-Qu conjugate NPs were similar to those of Ir-ss-Qu conjugate NPs without NR. The cell uptake of NR-loaded Ir-ss-Qu NPs and their intracellular drug release triggered by the reductive environment in MCF-7/ADR cells was analyzed by confocal laser scanning microscopy (CLSM). MCF-7/ADR cells were cultured with NR-loaded Ir-ss-Qu NPs for 3 h, and then they were observed using a LEICA TCS SP8. As shown in Figure 3C, the red fluorescence of NR was obviously observed in cells, which demonstrated that NR-loaded Ir-ss-Qu NPs could be internalized by the cells. In addition, the blue fluorescence was mainly located in the nucleus according to the merge image, which was ascribed to the rapid release of Ir from Ir-ss-Qu NPs by cleaving disulfide bonds under the higher intracellular concentration of GSH and resulted in the accumulation of Ir in the nucleus. The cellular uptake of NR-loaded Ir-ss-Qu NPs was further confirmed by flow cytometric analysis. The MCF-7/ADR cells were incubated with NR-loaded Ir-ss-Qu NPs for 1, 2, and 3 h. Then the fluorescence intensity of NR in cells was directly measured by flow cytometric analysis. As displayed in Figure 3D, the fluorescence intensity of cells enhanced gradually with the incubation time prolonged, which indicated more and more NR-loaded Ir-ss-Qu NPs could efficiently enter into MCF-7/ADR cells by cellular internalization.

### Studies of reversal MDR efficacy and mechanism *in vitro*

**Figure 4:**
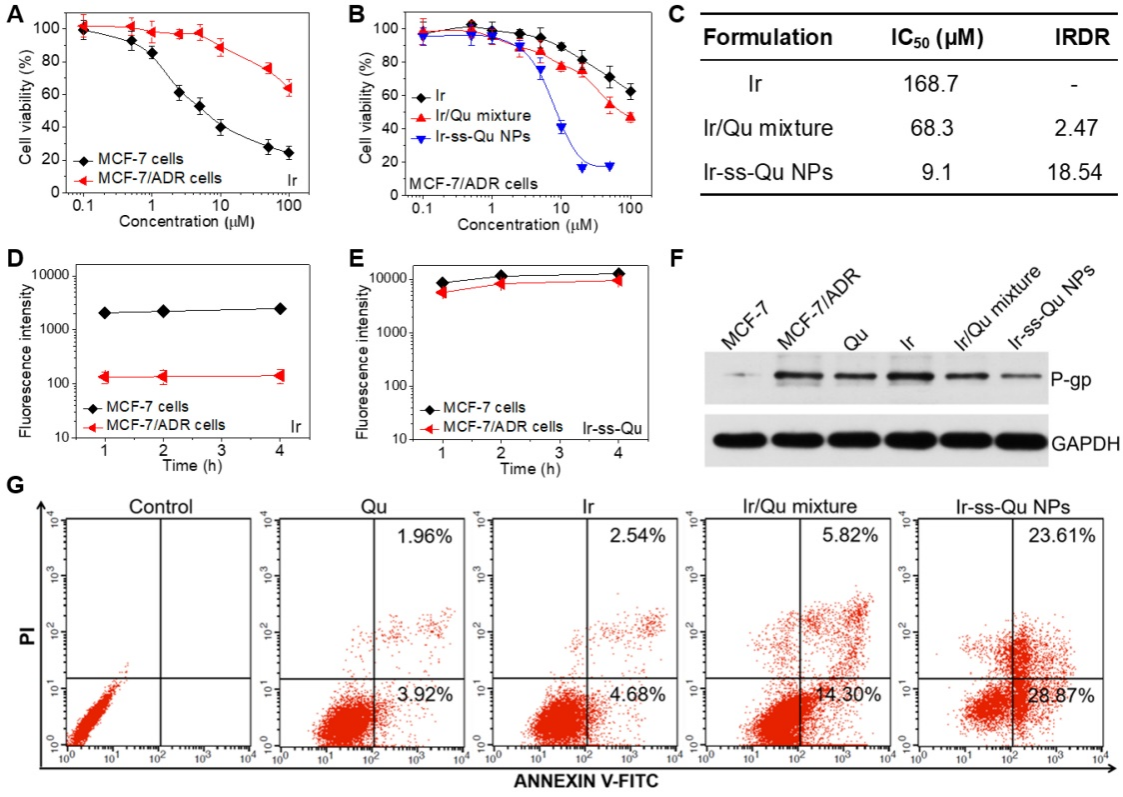
*In vitro* studies of reversal MDR efficacy.** (A**) *In vitro* cytotoxicity of Ir to MCF-7 cells and MCF-7/ADR cells determined by MTT assay. (**B**) *In vitro* cytotoxicity of Ir, Ir/Qu mixture and Ir-ss-Qu conjugate NPs to MCF-7/ADR cells determined by MTT assay. The data are presented as average standard error (n = 6). (**C**) The IC_50_ value and index of reversal drug resistance (IRDR) of different drug formulation against MCF-7/ADR cells. IRDR is calculated as the following equation: IRDR = IC_50_ (Ir to MCF-7/ADR cells) / IC_50_ (others drug formulations contain Ir to MCF-7/ADR cells). (**D**) The accumulation of free Ir in MCF-7 cells and MCF-7/ADR cells after incubation with Ir for different time. (**E**) The accumulation of Ir-ss-Qu in MCF-7 cells and MCF-7/ADR cells after incubation with Ir-ss-Qu conjugate NPs for different time. (**F**) The expression levels of P-gp in MCF-7/ADR cells induced by Qu, Ir, Ir/Qu mixture and Ir-ss-Qu conjugate NPs at the same concentration (20 μM) for 24 h, determined by western blot analysis. MCF-7 cells are used as a control, and GAPDH is the loading control. Data represent three individual experiments. Each experiment group is repeated three times. (**G**) Flow cytometry analysis for apoptosis of MCF-7/ADR cells induced by Qu, Ir, Ir/Qu mixture and Ir-ss-Qu conjugate NPs at the same concentration of 20 µM for 16 h. Lower left: living cells; Lower right: early apoptotic cells; Upper right: late apoptotic cells; Upper left: necrotic cells. Inserted numbers in the profiles indicate the percentage of the cells present in this area.

The *in vitro* cytotoxicity of Ir against MCF-7 and MCF-7/ADR cancer cells was evaluated by methyl tetrazolium (MTT) assay and the results exhibited in Figure 4A. The half-maximal inhibitory concentration values (IC_50_) of Ir for MCF-7 and MCF-7/ADR cells were 7.3 μM and 178.6 μM, respectively. The index of drug resistance (IDR) of MCF-7/ADR cells was calculated to be 24.47 μM (Table S1 in Supporting Information), suggesting that MCF-7/ADR cells were highly resistant to Ir. Therefore, MCF-7/ADR cells could be used as the MDR cells to study the reversal effect of Ir-ss-Qu NPs. The cytotoxicity of Qu towards MCF-7 and MCF-7/ADR cells was extremely low as shown in Figure S14, suggesting Qu was only the P-gp protein inhibitor and unable to inhibit the proliferation of cancer cells. The proliferation inhibition of Ir-ss-Qu NPs against MCF-7/ADR cells was investigated, comparing with free Ir, and Ir/Qu mixture by MTT assay. The cells without any treatment were used as the control. As shown in Figure 4B, the cytotoxicity to MCF-7/ADR cells of Ir/Qu mixture was slightly higher than that of free Ir. However, the cytotoxicity of Ir-ss-Qu was related to its concentration. When its concentration is lower than its CAC value, the anticancer activity of Ir-ss-Qu is the same as that of Ir/Qu mixture. If its concentration is higher than its CAC value, the therapeutic effect drastically increased and much higher than that of free Ir and Ir/Qu mixture, suggesting that Ir-ss-Qu NPs could effectively reverse the MDR of MCF-7/ADR cells to anticancer drug Ir. The IC_50_ values of different drug formulations against MCF-7/ADR cells were obtained according to the MTT assay (Figure 4B). As exhibited in the Figure 4C, the IC_50_ value of Ir in MCF-7/ADR cells was 168.7 μM, which indicated that the MDR of MCF-7/ADR cells to Ir was extremely serious. When the MCF-7/ADR cells were treated with Ir/Qu mixture, the IC_50_ value was slightly reduced to 68.3 μM with theindex of reversal drug resistance (IRDR) of 2.47. However, when the MCF-7/ADR cells were incubated with Ir-ss-Qu NPs, the IC_50_ value was remarkably decreased to 9.1 μM and the IRDR was up to 18.54. This further confirmed the drug formulation of Ir-ss-Qu NPs could efficiently reverse the MDR of Ir to MCF-7/ADR cells on the base of the synergistic effect of Ir and Qu rapidly released from Ir-ss-Qu NPs in the intracellular reductive conditions.

According to previous reports, Qu was able to inhibit the P-gp mediated efflux of drugs out of MDR cancer cells, which resulted in an increased intracellular accumulation of anticancer drugs.^39,40^ Hence, the effect of Qu on the intracellular accumulation and efflux of Ir was investigated by using MCF-7 cells and MCF-7/ADR cells to clarify the reversal MDR mechanism of Ir-ss-Qu NPs. As shown in Figure 4D, the accumulation of free Ir in MCF-7/ADR cells was extremely low even treated for 4 h due to the intensive action of MDR. In contrast, the accumulation of Ir (Ir-ss-Qu NPs) was relatively high and enhanced with the incubation time prolonged as a result of the low expression of P-gp. As predicted, the marked accumulation of Ir was detected in MCF-7/ADR cells as well as in MCF-7 cells when both of them were treated by Ir-ss-Qu NPs for the same time (Figure 4E). The amount of Ir in MCF-7/ADR cells was nearly the same as that in MCF-7 cells, which was attributed to the rapid release of Qu from Ir-ss-Qu NPs under the intracellular GSH to inhibit the expression of P-gp, which prevented Ir pumped out from MCF-7/ADR cells and resulted in the high intracellular accumulation of drugs. For drug efflux assay, after treated with free Ir and Ir-ss-Qu NPs for 4 h respectively, the cells were incubated with fresh medium and the retained amount of Ir was determined by BD LSRFortessa flow cytometer at different time interval. As shown in Figure S15A, when they were incubated with free Ir, the amount of Ir in MCF-7/ADR cells was very low and reduced with the subsequent treatments due to the efflux effect of P-gp. For MCF-7 cells, at the beginning period of measurement, the amount of Ir was significantly higher than that of MCF-7/ADR cells and no obvious decrease was observed in the whole process of incubation. However, when the MCF-7 cells and MCF-7/ADR cells were incubated with Ir-ss-Qu NPs, the concentration of Ir was still kept at a high level in both MCF-7 cells and MCF-7/ADR cells and no change was measured with prolonging the treatment time (Figure S15B), which was attributed to the similar reason as mentioned above. Owing to the MDR of MCF-7/ADR cells originated from the overexpression of P-gp on the cell surface, Western blot analysis was used to analyze the expression of P-gp. The MCF-7/ADR cells were treated separately with Qu, Ir, Ir/Qu mixture, and Ir-ss-Qu NPs at the same dose for 16 h. The MCF-7 cells and MCF-7/ADR cells without treatment were used as a control. As shown in Figure 4F, no significant expression of P-gp was tested in the MCF-7 cells. However, the expression of P-gp was extremely high in MCF-7/ADR cells nearly the same as treatment of Ir. After incubated with Qu and Ir/Qu mixture, the P-gp expression was downregulated slightly compared to that of the untreated MCF-7/ADR. The lower downregulated trend was attributed to the difficult uptake of hydrophobic Qu by MCF-7/ADR cells. When the MCF-7/ADR cells were incubated with Ir-ss-Qu NPs, the P-gp expression was remarkably downregulated, which indicated the strong effect of Ir-ss-Qu NPs on inhibition P-gp expression. The expression of P-gp was further determined by flow cytometer and the result was similar to that of Western blot analysis (Figure S16).

In general, cancer cells are killed by small molecule chemotherapeutic drugs through the activating apoptosis. The above discussion demonstrated that Qu was no cytotoxicity to cancer cells, but was able to increase the intracellular accumulation of Ir in MCF-7/ADR cells through inhibition of P-gp expression. Here the synergistic efficacy of Qu and Ir on MCF-7/ADR cells apoptosis was evaluated using FITC-Annexin V/propidium iodide (PI) method. Firstly, MCF-7/ADR cells were separately treated with Qu, Ir, Ir/Qu mixture, and Ir-ss-Qu NPs at the same concentration (20 μM) for 16 h and then stained with FITC-Annexin V/PI. The untreated MCF-7/ADR cells were used as control. The flow cytometry analysis results in Figure 4G shew that the ratio of apoptosis cells was 5.88%, 7.22%, or 20.12% induced by Qu, Ir, or Ir/Qu mixture, but enhanced up to 52.48% treated by Ir-ss-Qu NPs. This confirmed that Ir-ss-Qu NPs could promote a much higher apoptotic rate against MCF-7/ADR cells at the same concentration due to the synergistic effect of Ir and Qu released via cleavage of disulfide bond triggered by the intracellular reductive environment.

### Blood retention time and biodistribution *in vivo*

**Figure 5:**
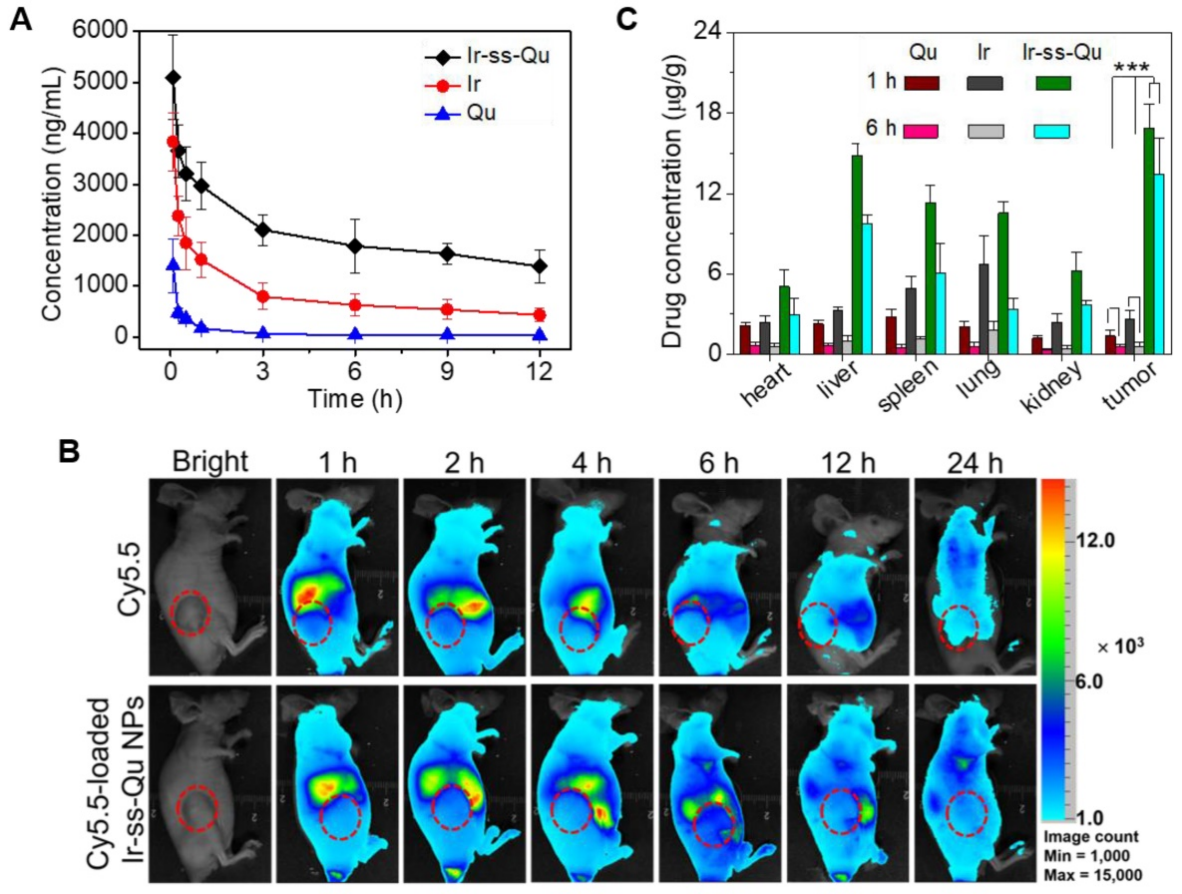
*In vivo* pharmacokinetics and blood retention time of Ir-ss-Qu conjugate NPs. (**A**) Representative plasma concentration-time profiles of free Qu, Ir and Ir-ss-Qu conjugate after i.v. injection into rats (a dose of 10 mg kg^-1^). The data are presented as the average standard error (n = 4). (**B**) *In vivo* non-invasive NIRF images of time-dependent whole body imaging of MCF-7/ADR tumor-bearing nude mice after intravenous injection of free Cy5.5 and Cy5.5-loaded Ir-ss-Qu conjugate NPs. (**C**) Tissue distribution of Qu, Ir, and Ir-ss-Qu after intravenous injection of free Qu (2.9 mg kg^-1^), Ir (5.3 mg kg^-1^), and Ir-ss-Qu NPs (10 mg kg^-1^) in nude mice. Data are presented as average ± standard error (n = 4), and the statistical significance level is ***P < 0.001.

It is well known that nanodrugs with a suitable size usually display a longer retention time compared with that of small molecule in the bloodstream *in vivo* 48. To verify this deduction, a pharmacokinetic study was evaluated by using Sprague-Dawley (SD) rats separately injected with free Qu, Ir, and Ir-ss-Qu NPs via tail vein. The time profiles of free Qu, free Ir, and Ir-ss-Qu NPs in plasma were shown in Figure 5A. Obviously, the concentration of free Qu or Ir significantly decreased to 25% in the blood within 3 h, whereas the concentration of Ir-ss-Qu remained at the higher level even after injection of 12 h. Such a longer blood retention time of Ir-ss-Qu NPs could facilitate them to accumulate in tumor tissues. To further determine the blood retention time of Ir-ss-Qu NPs, the MCF-7/ADR tumor-bearing nude mice were intravenously injected with a hydrophobic near- infrared fluorescence dye Cy5.5 or Cy5.5-loaded Ir-ss-Qu NPs (DLS date and TEM image in Figure S18), respectively. Subsequently, the real-time imaging of Cy5.5 or Cy5.5-loaded Ir-ss-Qu NPs in the tumor-bearing mice was monitored at predetermined time intervals. As shown in Figure 5B, after injection for 1 h, the strong fluorescence signal of Cy5.5 was detected in the whole mice body both Cy5.5 and Cy5.5-loaded Ir-ss-Qu NPs groups. However, the fluorescence signals of Cy5.5 in the whole body significantly decrease from 1 to 6 h in free Cy5.5-treated group. After injection of 24 h, the signals in the whole body were almost undetectable, suggesting that the free Cy5.5 had been rapidly cleared from the mice body. On the contrary, the signals of Cy5.5 in the whole body gradually increased up to 4 h after injection of Cy5.5-loaded Ir-ss-Qu NPs. Even upon injection for 24 h, the signals of Cy5.5 in Cy5.5-loaded Ir-ss-Qu treated group still kept in the higher intensity. The longer retention time definitely promoted the accumulation of nanodrugs in tumor tissue. Meanwhile, the *ex vivo* fluorescence images of tumors and organs isolated from the MCF-7/ADR tumor-bearing nude mice after intravenous injection for 24 h were shown in SI Figure S19 and the similar results were obtained, i.e., the stronger fluorescence signal of Cy5.5 was found in the Cy5.5-loaded Ir-ss-Qu NPs treated group. In addition, the biodistribution of Ir-ss-Qu NPs, Qu, and Ir in the tumors and normal organ tissues was investigated in MCF-7/ADR tumor model after intravenous injection with different time intervals. After 1 h injection, large amount of Ir-ss-Qu mainly accumulated in liver, spleen, lung, and tumor. When injection for 6 h, the quantity of Ir-ss-Qu conjugate obviously reduced in lung and spleen, nevertheless the content in tumor maintained at the higher level (Figure 5C). Moreover, the concentration of free Qu and Ir was extremely lower in the tumor compared to that of Ir-ss-Qu NPs, which suggested that the EPR effect resulted in the higher tumor accumulation of Ir-ss-Qu.

### Reversal effect of Ir-ss-Qu NPs for MCF-7/ADR in-duced xenografts *in vivo*

**Figure 6:**
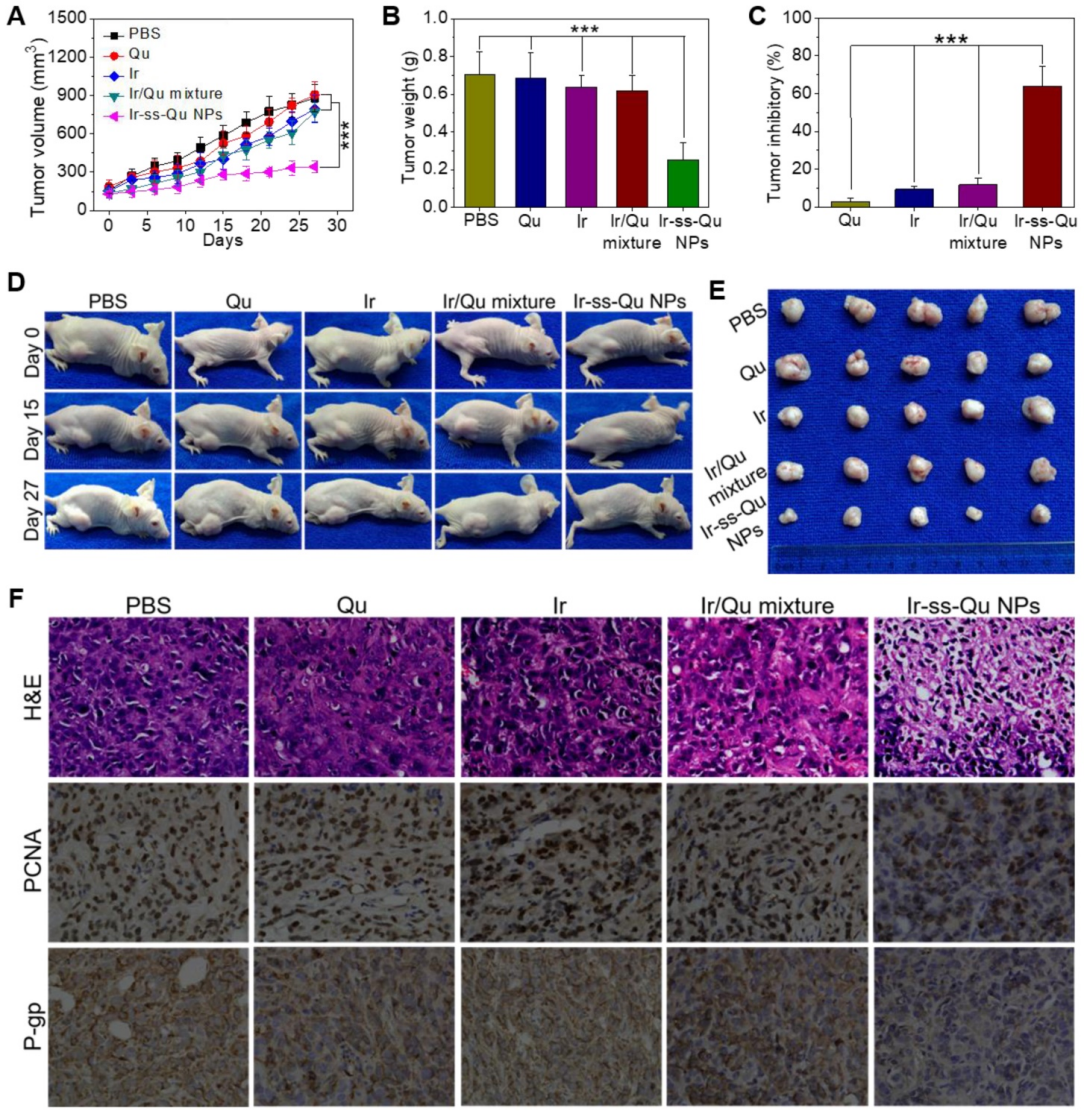
*In vivo* reversal activity assay. (**A**) Changes of tumor volume after intravenous injection of PBS, Qu, Ir, Ir/Qu mixture and Ir-ss-Qu conjugate NPs in MCF-7/ADR tumor-bearing nude mice. (**B**) Mean weight of tumors separated from mice after different treatments. (**C**) The tumor inhibitory rate (TIR) after treated with PBS, Qu, Ir, Ir/Qu mixture and Ir-ss-Qu conjugate NPs in MCF-7/ADR tumor-bearing nude mice. The TIR is calculated using the following equation: TIR = 100% × (mean tumor weight of control group ‒ mean tumor weight of experimental group)/mean tumor weight of control group. Data are represented as average standard error (n = 6). Statistical significance: **P < 0.005; ***P < 0.001. (**D**) The mice are treated with different formulations and the tumor size is real-time monitored during the 27-day evaluation period. (**E**) Representative tumors separated from animals after intravenous injection of PBS, Qu, Ir, Ir/Qu mixture and Ir-ss-Qu conjugate NPs. (**F**) H&E, PCNA expression and P-gp photographs of tumor tissues of MCF-7/ADR tumor-bearing mice after being treated by intravenous injection of PBS, Qu, Ir, Ir/Qu mixture, and Ir-ss-Qu conjugate NPs for 27 days (magnification × 400).

The reversal activity of Ir-ss-Qu NPs was evaluated by using MCF-7/ADR cells xenograft models and compared to that of free Qu, Ir, and Ir/Qu mixture. All of drug formulations were separately injected into MCF-7/ADR tumor-bearing mice via the tail vein every 3 days for 27 days, and physiological saline as a control. As shown in Figure 6A, no significant tumor inhibitory effect was observed in Qu group, which confirmed further Qu was only P-gp inhibitor. After the treatment was completed, the tumor volume of free Ir and Ir/Qu group was 89.90 ± 6.91% and 87.43±4.64% of control group, respectively. There was no obvious differ-ence of antitumor efficacy between the groups treated with Ir or Ir/Qu mixture, which indicated that Qu could not promote the therapeutic activity of Ir *in vivo* by simply physical mixing. However, the tumor volume of Ir-ss-Qu NPs group was 38.92 ± 2.53% at the end of treatment, which indicated that Ir-ss-Qu NPs achieved the highest antitumor effect comparing with that of Qu, Ir and Ir/Qu mixture. Moreover, the body weights of all mice groups were kept at a stable level during the whole process of treatment, which illustrated the safety of Ir-ss-Qu NPs (Figure S20). After 27 days treatment, MCF-7/ADR tumor-bearing mice were sacrificed and tumors were dissected, washed, photographed (Figure 6E), and weighted (Figure 6B) for calculating the tumor inhibitory rate (TIR) as shown in Figure 6C. The TIR of Ir-ss-Qu NPs was 64.10 ± 8.27% and remarkably higher than that of Qu (2.68 ± 1.06%), Ir (9.22 ± 3.46%) and Ir/Qu mixture (11.79 ± 4.28%). In addition, the tumor growth throughout the treatment course was observed at the pre-determined time by taking photographs (Figure 6D). Compared to that treated with the PBS, Qu, Ir, and Ir/Qu mixture groups, the tumor size of Ir-ss-Qu NPs-treated group was almost no obvious variation after 27 days, which demonstrated that Ir-ss-Qu NPs could effectively reverse the MDR of MCF-7/ADR tumor. The excellent therapeutic efficacy of Ir-ss-Qu NPs was attributed to the advantage of nanodrugs, including the high tumor drug accumulation, facilitated cellular uptake of drugs, and the synergistic action of Ir and Qu released in MCF-7/ADR cells triggered by GSH.

To further evaluate the antitumor efficacy after treatment with various formulations, the tumor tissues were dissected from mice and sectioned for the histological and immunohistochemical analysis. As shown in Figure 6F, the hematoxylin and eosin (H&E) assay displayed that the dense tumor cells with large nucleus and spherical shape were observed in tumor tissue treated with PBS, which meant a rapid growth of MCF-7/ADR tumor. The same results were obtained from the treatments with Qu, Ir, and Ir/Qu mixture. However, the number of tumor cells reduced observably, and nuclear shrinkage and even extensive cell fragmentation were observed in the group treated by Ir-ss-Qu NPs. Furthermore, the cell proliferating in MCF-7/ADR tumor tissues treated by various formulations was analyzed by immunhistochemical staining of the proliferation cell nuclear antigen (PCNA). The percentage of PCNA-positive (brown) tumor cells was no difference in tumor tissue treated by Qu, Ir, and Ir/Qu compared with that treated by PBS. Contrarily, the percentage of PCNA-positive tumor cells treated by Ir-ss-Qu NPs decreased significantly in tumor tissue, which was ascribed to the inhibition of cell proliferation in MCF-7/ADR tumor. Additionally, the in situ P-gp assay indicated that the expression of P-gp was obviously high in PBS- and Ir-treated group because of no inhibitor Qu in the treatment. When the therapeutic group contained Qu, such as Qu, Ir/Qu, and Ir-ss-Qu NPs, the expression of P-gp was inhibited efficiently in MCF-7/ADR tumor tissue. Among them, the P-gp inhibition activity of Ir-ss-Qu NPs was the best one because of the more accumulation of Qu in tumor site via the self-delivery of Ir-ss-Qu NPs. All above results confirmed the superior reversal effect of Ir-ss-Qu NPs for MDR *in vivo*. The biological safety was another major concern for application of chemotherapy *in vivo*. The main organs, including heart, liver, spleen, lung, and kidney, were handled by H&E staining method to evaluate the biosecurity of various formulation as shown in Supporting Information Figure S21. Compared to those of PBS group, all the organs of Ir-ss-Qu NPs group exhibited relatively normal histological structures, which verified the low systemic toxicity of Ir-ss-Qu NPs and potential application for cancer clinical therapy.

## Conclusion

In summary, we designed and fabricated a redox-responsive amphiphilic drug-inhibitor conjugate (ADIC) via a linkage containing cleavable disulfide bond for overcoming the MDR of tumor. It was able to self-assemble into NPs in aqueous solution. The Ir-ss-Qu NPs were very stable in physiological environment and exhibited the longer retention time than that of free Ir in the bloodstream, which facilitated the accumulation of Ir-ss-Qu NPs in tumor tissues. After they were uptaken by MCF-7/ADR cells, Ir and Qu rapidly released from Ir-ss-Qu NPs via cleavage of disulfide bond under the intracellular GSH reductive action. The released Qu significantly down-regulated the expression of P-gp and efficiently prevented Ir to be pumped out of the MCF-7/ADR cells and resulted in the higher accumulation of Ir in the cells, which exhibited the higher apoptotic rate and superior *in vitro* and *in vivo* reversal MDR activity than those of free Ir. Overall, we believe that this strategy based on redox- responsive amphiphilic drug-inhibitor conjugate may offer a new way for overcoming the MDR of tumor and eventually improve the success rate of chemotherapy in clinic.

## Materials and Methods

### Materials

*N*,*N*'-Dicyclohexylcarbodiimide (DCC, 99%, J& K), quinine (Qu, 99%, J&K), 4-(dimethylamino)pyridine (DMAP, 99%, J&K), 4,4'-dithiodibutyric acid (DTBA, 98%, J&K) pyrene (98%, J&K), 3-(4,5- dimethyl-thiazol-2-yl)-2,5-diphenyl tetrazolium bromide (MTT, Sigma), nile red (NR, 99%, Sigma- Aldrich), cyanine (Cy5.5, 99%, Fanbo) were used as received without further purification. Irinotecan (Ir) was purchased from Shanghai Knowshine Pharmachemical Inc. Alexa fluor® 488 annexin V/dead cell apoptosis assay kit was purchased from Invitrogen and used as received. GAPDH polyclonal antibody and rabbit anti-P-gp monoclonal antibody were purchased from Cell Signaling Technology (Danvers, MA, USA). All secondary antibodies were obtained from Earthox LLC. (Earthox, USA). Chloroform (CHCl_3_) was dried by refluxing with calcium hydride and distilled just before use. All other reagents and solvents were purchased from the domestic suppliers and used as received.

### Characterization

^1^H and ^13^C nuclear magnetic resonance spectroscopy (^1^H NMR and ^13^C NMR) spectra were recorded using a Varian Mercury Plus 400 MHz spectrometer with deuterium chloroform (CDCl_3_) as a solvent. LC-MS was performed on a Water ACQUITY UPLC system equipped with a binary solvent delivery manager and a sample manager, coupled with a Waters Q-TOF Premier Mass Spectrometer equipped with an electrospray interface (Waters Corporation, Milford, MA). MS analysis was carried out via Masslynx 4.1 software (Waters MS Technologies, Manchester, UK). FTIR spectra were recorded on a Paragon 1,000 instrument by KBr sample holder method. UV-Vis absorption of the sample solutions was measured at room temperature by using a Thermo Electron-EV300 UV-Vis spectrophotometer. The slit-width was set as 1 nm with a scan speed of 480 nm min^-1^. Fluorescent spectra were recorded on QC-4-CW spectrometer, made by Photon technology international, Int. USA/CAN. The excitation wavelength was set at 360 nm, which was chosen according to the maximum intensity obtained in the excitation spectra. Step increment was set as 2 nm, and scan speed was set at 480 nm min^-1^. DLS measurements were performed under a Malvern Zetasizer 3,000 HS (Malvern Instruments, Ltd.) equipped with a 125 mW laser light and operated at λ = 633 nm. All samples were measured at a scattering angle of 90o. TEM studies were performed with a JEOL 2010 instrument operated at 200 kV. A little drop of the sample solution (0.5 mg mL^-1^) was sprayed onto the carbon-coated copper grid. Then, the grid was immersed into liquid nitrogen and frozen-dried in vacuum at -50 °C before measurement.

### Synthesis of Qu-ss-COOH

Typically, 4,4′-dithiobutyric acid (DTBA, 4.77 g, 20 mmol), DCC (2.27 g, 11 mmol), DMAP (1.22 g, 10 mmol) and THF (100 mL) were charged in a 250 mL round-bottle flask, stirred for 30 min at room temperature. And then Qu (3.24 g, 10 mmol) in 50 mL THF was added dropwise to the reaction mixture. After the addition was completed, the reaction mixture was stirred at room temperature for 24 h. After filtration and evaporation all the solvent, the crude product was purified by silica gel column chromatograph using CH_2_Cl_2_/CH_3_OH (20/1, v/v) as the eluent. The product was collected and the solvent was removed by rotary evaporation to give a white solid (3.80 g, yield: 69.7%). ^1^H NMR (400 MHz, CDCl_3_) δ (ppm): 10.62 (s, 1H), 8.70 (d, *J* = 4 Hz, 1H), 8.01 (d, *J* = 8 Hz, 1H), 7.46 (d, *J* = 2 Hz, 1H), 7.38 (dd, *J* = 2 Hz, *J* = 2 Hz, 1H), 7.25 (s, 1H), 6.81 (s, 1H), 5.65 (m, 1H), 4.99 (t, *J* = 28 Hz, 2H), 4.00 (s, 3H), 3.48 (s, 1H), 3.39 (s, 1H), 3.23 (t, *J* = 24 Hz, 1H), 2.91 (t, *J* = 24 Hz, 1H), 21.70 (m, 1H), 2.55 (m, 1H), 2.46 (s, 1H), 2.36 (t, *J* = 16 Hz, 2H), 2.08 (m, 2H), 1.94 (m, 4H), 1.82 (m, 1H), 1.72 (m, 1H), 1.62 (m, 1H). ^13^C NMR (100 MHz, CDCl_3_) δ (ppm): 177.55, 171.57, 158.89, 147.04, 144.48, 142.59, 139.67, 131.64, 126.44, 122.89, 117.94, 116.16, 101.17, 72.18, 58.01, 56.44, 55.27, 42.86, 38.41, 37.74, 34.01, 32.48, 27.53, 26.37, 25.12, 23.76, 22.01. ESI-MS m/z (M+H^+^) calcd 545.2144, found 545.2158 (M+H^+^).

### Synthesis of Ir-ss-Qu conjugate

Typically, Qu-ss-COOH (2.32 g, 4.26 mmol), Ir (2.26 g, 3.85 mmol), DCC (1.03 g, 5 mmol), DMAP (0.43 g, 3.5 mmol) and CH_2_Cl_2_ (100 mL) were charged in a 250 mL round-bottle flask, stirred for 24 h at room temperature. After filtration and evaporation all the solvent, the crude product was purified by silica gel column chromatograph using CH_2_Cl_2_/CH_3_OH (20/1, v/v) as the eluent. The product was collected and the solvent was removed by rotary evaporation to give a yellowy solid (3.26 g, yield: 76.1%). ^1^H NMR (400 MHz, CDCl_3_) δ (ppm): 8.71 (d, *J* = 4 Hz, 1H), 8.19 (d, *J* = 12 Hz, 1H), 7.99 (d, *J* = 8 Hz, 1H), 7.83 (d, *J* = 4 Hz, 1H), 7.58 (dd, *J* = 4 Hz, J = 4 Hz, 1H), 7.42 (d, *J* = 4 Hz, 1H), 7.35 (d, *J* = 4 Hz, *J* = 4 Hz, 1H), 7.32 (d, *J* = 4 Hz, 1H), 7.15 (s, 1H), 6.48 (d, *J* = 4 Hz, 1H), 5.82 (m, 1H), 5.67(d, *J* = 16 Hz, 1H), 5.40 (d, *J* = 16 Hz, 1H), 5.23 (d, *J* = 4 Hz, 2H), 5.01 (m, 2H), 4.40 (dd, *J* = 16 Hz, *J* = 12 Hz, 2H), 3.94 (s, 3H), 3.34 (m, 1H), 3.09 (m, 6H), 2.90 (t, *J* = 24 Hz, 1H), 2.62 (m, 12H), 2.50 (m, 2H), 2.25 (m, 2H), 2.13 (m, 2H), 1.99 (m, 6H), 1.87 (m, 3H), 1.69 (m, 7H), 1.49 (m, 4H), 1.38 (t, *J* = 16 Hz, 3H), 0.96 (t, *J* = 16 Hz, 3H). ^13^C NMR (100 MHz, CDCl_3_) δ (ppm): 172.24, 172.11, 167.76, 158.12, 157.54, 153.28, 151.62, 150.64, 147.63, 147.29, 147.07, 146.09, 145.56, 144.91, 143.79, 141.84, 131.96, 131.74, 127.75, 127.29, 127.17, 126.18, 122.07, 120.07, 119.09, 114.79, 101.57, 95.96, 76.17, 73.99, 67.31, 62.58, 59.31, 56.73, 55.92, 50.47, 49.50, 44.58, 44.25, 42.68, 39.82, 37.54, 37.27, 32.77, 32.32, 31.99, 28.40, 27.91, 27.70, 26.24, 24.72, 24.58, 24.13, 23.38, 14.23, 7.81. ESI-MS m/z (M+H^+^) calcd 1113.4830, found 1113.4860 (M+H^+^).

### Preparation of Ir-ss-Qu conjugate nanoparticles

Briefly, 5 mg Ir-ss-Qu conjugate was dissolved in 1 mL of dimethylsulfoxide (DMSO) and stirred at room temperature for 2 min. After the Ir-ss-Qu conjugate dissolved completely, the solution was slowly added to 3 mL of deionized water and stirred slightly for 10 min. Subsequently, the solution was dialyzed against deionized water for 24 h (molecular weight cutoff = 3,500 g mol^-1^), during which the water was renewed every 4 h. The volume of the solution was increased to 10 mL with the addition of deionized water to produce a solution with a concentration of 0.5 mg mL^-1^ for further experiments.

### Critical aggregation concentration (CAC) measurement

To determine the CAC value of the Ir-ss-Qu conugate, pyrene was used as the fluorescence probe. 2 µL of pyrene acetone solution (6 × 10^-4^ mol L^-1^) was added to 2 mL of Ir-ss-Qu conugate aqueous solution with different concentrations, while the concentration of pyrene in each flask was kept at 6 × 10^-7^ mol L^-1^. The fluorescence emission spectra of all samples were recorded on a LS-50B luminescence spectrometer (Perkin Elmer Co.) at 335 nm excitation wavelength and 8 nm slit width. The *I*_3_/*I*_1_ values of all solution were recorded.

### Cellular uptake of Ir-ss-Qu conjugate nanoparticles by MCF-7/ADR cells

The cellular uptake behaviors were evauted in MCF-7/ADR cells using confocal laser scanning microcopy (CLSM) and flow cytometry. For the CLSM study, MCF-7/ADR cells were seeded in 6-well plates at 2.0 × 10^5^ cells per well in 1 mL of complete DMEM and incubated for 24 h, followed by removing culture medium and adding NR-loaded Ir-ss-Qu conjugate nanoparticle solutions (0.5 mL DMEM medium) at the concentration of 30 µM. After incubation at 37 °C for 6 h, culture medium was removed, and cells were washed with PBS for two times. Subsequently, the cells were fixed with 4% formaldehyde for 30 min at room temperature, and the slides were rinsed with PBS three times. The resulting slides were mounted and observed with a LEICA TCS SP8 fluorescence microscopy. For flow cytometry, MCF-7/ADR cells were seeded in 6-well plates at 5.0 × 10^5^ cells per well in 2 mL of complete DMEM and cultured for 24 h. Then the solution of NR-loaded Ir-ss-Qu conjugate nanoparticles was diluted with DMEM culture medium at a final concentration of 30 µM. The diluted solution was added to different wells and the cells were incubated at 37 °C for 1, 2, and 3 h. Thereafter, culture medium was removed and cells were washed with PBS for three times and treated with trypsin. Data for 1.0 × 10^4^ gated events were collected and analysis was performed by means of a BD LSRFortessa flow cytometer.

### *In vitro* cytotoxicity assay

For the cytotoxicity of Qu and Ir to MCF-7 and MCF-7/ADR cells: The cells were seeded into 96-well plates at 8.0 × 10^3^ cells per well in 200 µL of DMEM. After 12 h incubation, the medium was removed and replaced with 200 µL of a medium containing serial dilutions of inhibitor Qu from 0.1 to 50 µM and the serial dilutions of Ir from 0.1 to 100 µM. The cells without the treatment were used as control. The cells were grown for another 72 h. Then, 20 μL of MTT solution (5 mg mL^-1^) in PBS was added to each well. After the cells were incubated for 3.5 h, the medium containing unreacted MTT was carefully removed. Then, the obtained blue formazan crystals were dissolved in 200 μL well^-1^ DMSO, and the absorbance was measured in a BioTek Synergy H4 hybrid reader at a wavelength of 490 nm. The blank was subtracted to the measured optical density (OD) values, and the cell viability was expressed as % of the values obtained for the untreated control cells.

For the cytotoxicity of Ir, Ir/Qu mixture and Ir-ss-Qu conjugate nanoparticles to MCF-7/ADR cells: The cells were seeded into 96-well plates at 8.0 × 10^3^ cells per well in 200 µL of DMEM. After 12 h incubation, the medium was removed and replaced with 200 µL of a medium containing serial dilutions of Ir and Ir/Qu mixture from 0.1 to 100 µM and the serial dilutions of Ir-ss-Qu conjugate nanoparticles from 0.1 to 50 µM. The cells without the treatment were used as control. The cells were grown for another 72 h. Then, 20 μL of MTT solution (5 mg mL^-1^) in PBS was added to each well. After the cells were incubated for 3.5 h, the medium containing unreacted MTT was carefully removed. Then, the obtained blue formazan crystals were dissolved in 200 μL well^-1^ DMSO, and the absorbance was measured in a BioTek Synergy H4 hybrid reader at a wavelength of 490 nm.

### Cell apoptosis assay

MCF-7/ADR cells were seeded in 6-well plates at 5.0 × 10^5^ cells per well in 2 mL of complete DMEM and cultured overnight. The cells were treated with Qu, Ir, Ir/Qu mixture and Ir-ss-Qu conjugate nanoparticles at the same concentration (20 µM) for 16 h. MCF-7/ADR cells without the treatment were used as a control. For quantitative measurement of apoptosis, treated cells were harvested and washed twice with ice-cold PBS, stained with Alexa Fluor® 488 annexin V and PI according to the manufacturer's instructions.

### Pharmacokinetics and biodistribution

For pharmacokinetic studies, SD rats (~ 200 g) were randomly divided into Ir-ss-Qu conjugate nanoparticles and free Qu, Ir groups (n = 4). The aqueous solutions of Ir-ss-Qu conjugate nanoparticles and free Qu and Ir were intravenously injected via tail vein at a dose of 10 mg kg^-1^. The blood samples (0.5 mL) were taken from the eye socket at the 5 min, 15 min, 30 min, 1 h, 3 h, 6 h, 9 h, and 12 h time points after injection. The plasma was obtained by centrifugation at 3,000 rpm for 10 min and stored at -20 °C. We treated 200 µL of plasma two times with 300 µL of dichloromethane. The solvent solutions separated by centrifugation were pooled. The samples of Ir-ss-Qu and free Ir were directly examined by using fluorescence spectroscopy, and the samples of free Qu were measured by use of LC. The amounts of Ir-ss-Qu conjugate, free Qu and Ir were obtained from standard curves previously obtained by analysis of blood samples containing known amounts of Ir-ss-Qu, Qu and Ir.

To assess the tissue distribution of free Qu, Ir and Ir-ss-Qu conjugate nanoparticles, the MCF-7/ ADR tumor-bearing mice were intravenously injected via tail vein with Qu, Ir and Ir-ss-Qu conjugate nanoparticles at a dose of Qu (2.9 mg kg^-1^), Ir (5.3 mg kg^-1^) and Ir-ss-Qu conjugate nanoparticles (10 mg kg^-1^). Mice were sacrificed by cervical vertebra dislocation at 30 min, 1 h and 6 h after drug administration (n = 3 at each time point), and the heart, liver, spleen, lung, kidney and tumor were collected. Tissue samples were rinsed in saline, blotted using paper towel, weighed and stored at -80 °C before being homogenized. Qu, Ir and Ir-ss-Qu were extracted from the homogenate using 2 mL of dichloromethane. The organic phases were collected and dried, and the samples were dissolved in acetonitrile for analysis. The samples of Ir-ss-Qu and free Ir were directly examined by using fluorescence spectroscopy, and the samples of free Qu were measured by use of LC. The amounts of Ir-ss-Qu conjugate, Qu, and Ir were obtained from standard curves previously obtained by analysis of tissues samples containing known amounts of Ir-ss-Qu, Qu and Ir.

### *In vivo* anticancer activity

The MCF-7/ADR tumor-bearing mice were randomly divided into five groups, and mice in different treatment groups were intravenously injected via the tail vein with PBS, Qu (2.9 mg kg^-1^), Ir (5.3 mg kg^-1^), Ir/Qu mixture (2.9 mg kg^-1^ Qu and 5.3 mg kg^-1^ Ir) and Ir-ss-Qu conjugate nanoparticles (10 mg kg^-1^) once every 3 days for 27 days. Each mouse of different group was earmarked and followed individually throughout the whole experiments. The length and width of the tumor and the body weight of mice were measured before every injection by the end of experiment. Tumor volume (V) was calculated using the formula: V (mm^3)^ = 1/2 × length (mm) × width (mm)^2^. After 27 days postinjection, mice were sacrificed, and tumors were separated, weighted and photographed. In addition, the tumors were cut into small pieces, fixed in 10% formalin and embedded in paraffin. Then the tissues embedded in paraffin were sectioned for histopathological analysis with H&E staining.

### Statistical analysis

All data presented are Mean ± SD. The statistical analysis was assessed using a Student's t-test. The differences between experimental groups and control groups were considered statistically significant for ***P value < 0.01.
