# Supplementary material for: Stimuli-responsive nanodrug self-assembled from amphiphilic drug-inhibitor conjugate for overcoming multidrug resistance in cancer treatment
Source: Theranostics. 2019 Aug 12;9(20):5755–68. doi: 10.7150/thno.36163 (PMC6735370; doi:10.7150/thno.36163)
Supplement: Supplementary file 1 — Supplementary figures and tables. [file thnov09p5755s1.pdf]

Supporting Information

Stimuli-responsive nanodrug self-assembled from amphiphilic drug-inhibitor conjugate for overcoming multidrug resistance in cancer treatment

Ping Huang[1,2,†], Guanchun Wang[1,†], Yue Su[1], Yongfeng Zhou[1], Wei Huang[1,✉], Rong Zhang[2,✉], Deyue Yan[1,✉]

[1]School of Chemistry and Chemical Engineering, State Key Laboratory of Metal Matrix Composites, Shanghai Jiao Tong University, 800 Dongchuan Road, Shanghai 200240, P. R. China

[2]Department of Obstetrics and Gynecology, Fengxian Hospital, Southern Medical University, Shanghai 201499, China

[†]These authors contributed equally to this work.

***Corresponding author:**

Tel: +86-21-54742664; Fax: +86-21-54741297

E-mail address: dyyan@sjtu.edu.cn (D. Y. Yan); hw66@sjtu.edu.cn (W. Huang); rongzhang@163.com (R. Zhang)

Method

GSH-triggered *In vitro* Ir release from Ir-ss-Qu conjugate nanoparticles

Typically, 2 mL of Ir-ss-Qu conjugate nanoparticles (0.5 mg mL^{-1}) was transferred into a membrance tubing (MWCO = 3,500). It was incubated at 37 °C in 50 mL PBS (pH 7.4) containing (or not) 1 mM or 5 mM GSH at predetermined time intervals. Then 3 mL of external buffer solution was withdraw and replaced with 3 mL of fresh PBS (pH 7.4) containing (or not) 1 mM or 5 mM GSH. The amount of released Ir was determined by using fluorescence measurement (QC-4-CW spectrometer, excitation at 360 nm).

Preparation of NR-loaded/Cy5.5-loaded Ir-ss-Qu conjugate nanoparticles

In a typical procedure: 5 mg Ir-ss-Qu conjugate was dissolved in 1 mL of DMSO, followed by adding a 0.2 mL of NR solution (0.4 mg mL^{-1}) or 0.2 mL of Cy5.5 solution (1 mg mL^{-1}) in DMSO and stirred at room temperature for 10 min. Then the mixture was slowly added into 3 mL of deionized water and stirred slightly for another 10 min. Subsequently, the solution was dialyzed against deionized water for 16 h (molecular weight cutoff = 1,000 g mol^{-1}) and the deionized water was exchanged for 4 times. In order to determine the amount of NR or Cy5.5, the NR-loaded or Cy5.5-loaded Ir-ss-Qu conjugate nanoparticle solution was lyophilized and then dissolved in DMSO again. The NR or Cy5.5 content in Ir-ss-Qu conjugate nanoparticles was determined by the absorbance at 655nm for NR or 680 nm for Cy5.5 measured using an UV/Vis spectrophotometer.

Cell culture

MCF-7 cells and MCF-7/ADR cells were cultured in Dulbecco's Modified Eagle's medium (DMEM). The culture mediums contain 10% fetal bovine serum (FBS) and antibiotics (50 units mL^{-1} penicillin and 50 units mL^{-1} streptomycin) at 37 °C under a humidified atmosphere containing 5% CO_2.

The accumulation and efflux assay of Ir-ss-Qu conjugate nanoparticles

For drug accumulation assay, MCF-7 cells and MCF-7/ADR cells were seeded in 24-well plates at a density of 5×10^4 per well in 0.5 mL of complete DMEM and incubated overnight. Then the cells were treated with free Ir and Ir-ss-Qu conjugate nanoparticles for 1, 2 and 4 h at the same concentration (20 μM) at 37 °C. At the end of experiment, the cells were washed for three times with ice-cold PBS and trypsinized, resuspended in 500 μL PBS. Data for 1.0×10^4 gated events were collected and analysis was performed by means of a BD LSRFortessa flow cytometer. The fluorescent intensity was calculated by CellQuest software, and blanked by untreated cells.

For drug efflux assay, MCF-7 cells and MCF-7/ADR cells were first cultured with free Ir and Ir-ss-Qu conjugate nanoparticles at the same concentration (20 μM) at 37 °C for 4 h. Then, the medium was removed and the cells were washed with cold PBS for twice, followed by incubation with fresh medium for 1, 2, and 4 h. The amounts of Ir and Ir-ss-Qu in cells were determined by BD LSRFortessa flow cytometer.

P-gp assay

MCF-7/ADR cells (5.0×10^5 cell/well) were seeded in 6-well plates and cultured

for 24 h. The cells were treated with Qu, Ir, Ir/Qu mixture and Ir-ss-Qu conjugate nanoparticles at the same concentration (20 µM) at 37 °C for 24 h. MCF-7/ADR cells incultured in complete DMEM were used as control. Then, cells were trypsinized with trypsin-EDTA solution and collected with centrifugation at 800 rpm for 5 min. Subsequently, the cells were rinsed with cold PBS and resuspended in 0.5 mL PBS for flow cytometry analysis. The phycoerythrin (PE)-antihuman MDR1 (CD243, P-gp, ABCB1, eBioscience, USA) was used to label the P-gp on cell surface according to the manufacture's instruction, while the nonspecific labeling was corrected by PE-Mouse IgG2a (eBioscience, USA). Then, the P-gp expression were measured with flow cytometry by monitoring the fluorescent intensity and analyzed with the CellQuest software.

Western blot analysis

MCF-7/ADR cells were seeded in 6-well plates at a density of 1.0×10^6 cells per well in 2 mL of complete DMEM and allowed to culture for 24 h. The cells were treated with Qu, Ir, Ir/Qu mixture and Ir-ss-Qu conjugate nanoparticles at the same concentration (20 µM) for 16 h. MCF-7/ADR cells untreated were used as a negative control. After treatment for 16 h, the MCF-7/ADR cells were harvested. The cellular proteins were extracted in Laemmli buffer and the protein content in the extracts was quantified using a bicinchoninic acid (BCA) protein assay kit (Pierce, USA). Equal amounts of proteins (30 µg $lane^{-1}$) were separated on sodium dodecyl sulfate-polyacrylamide gels (SDS-PAGE) and electrotransferred to 0.22 µm polyvinylidene fluoride (PVDF) membranes. The membranes were then blocked with

5% non-fat dry milk in TBST (Tris buffered saline supplemented with 0.05% Tween-20) and probed with antibodies against GAPDH (1:500 dilution), P-gp (1:500 dilution) followed by HRP-conjugated (HRP: horseradish peroxidase) anti-rabbit immunoglobulin-G (IgG, 1:5,000 dilution). GAPDH was used as the loading control. Protein bands were detected using Chemiluminescent HRP Substrate (Themo Scientific, USA) according to the manufacture's protocol and analyzed using the ChemiDocTM MP Imaging System (Bio-Rad, USA).

Animals and tumor models

Study protocols involving animals were approved by the Animal Ethics Committee of Shanghai Jiao Tong University School of Medicine. SD rats (~ 200 g) and 72 Balb/c female nude mice (4 weeks of age) were supplied by Chinese Academy of Sciences (Shanghai). The female nude mice were injected subcutaneously in the right flank region with 200 μL of cell suspension containing 1.0×10^7 MCF-7/ADR cells. The tumors were allowed to grow to ~ 100 mm^3 before experimentation.

***In vivo* optical imaging**

MCF-7/ADR tumor-bearing mice were randomly separated into two groups, and injected intravenously via tail vein with 200 μL of Cy5.5-loaded Ir-ss-Qu conjugate nanoparticles and free Cy5.5 possessed similar absorption intensity of Cy5.5. The fluorescence distribution was monitored at 1, 2, 4, 6, 12, and 24 h using an *in vivo* imaging system with appropriate wavelength (ex = 690 nm, em = 700 nm).

Immunohistochemical analysis

3 days after the last injection, mice were sacrificed and tumor tissues were excised.

The tissues were fixed in 10% formalin and embedded in paraffin. The paraffin-embedded 5 μm tumor sections were analyzed by immunohistochemical analysis for PCNA and P-gp protein expression. Firstly, endogenous peroxidase activity was inhibited by 3% hydrogen peroxide aqueous solution for 10 min, and the sections were heated to boiling in 0.01 M sodium citrate buffer (pH 6.0) for 10 min in the microwave oven for antigen retrieval and repeated boiling process for once. Subsequently, the sections were allowed to cool in the same buffer, rinsed twice with PBS for 5 min and then incubated with PCNA and P-gp antibody (Boster, China, 1:200) for 30 min at room temperature respectively and then at 4 ^{o}C overnight. After washing, sections were incubated with biotinylated secondary antibodies for 30 min at 37 °C. Finally, a streptavidin-biotin complex was applied and the immunoreactivity was visualized with diaminobenzidine as a chromogen. The sections were imaged by using Olympus Fluorescence Microscope (Olympus BX61).

Figures and Table

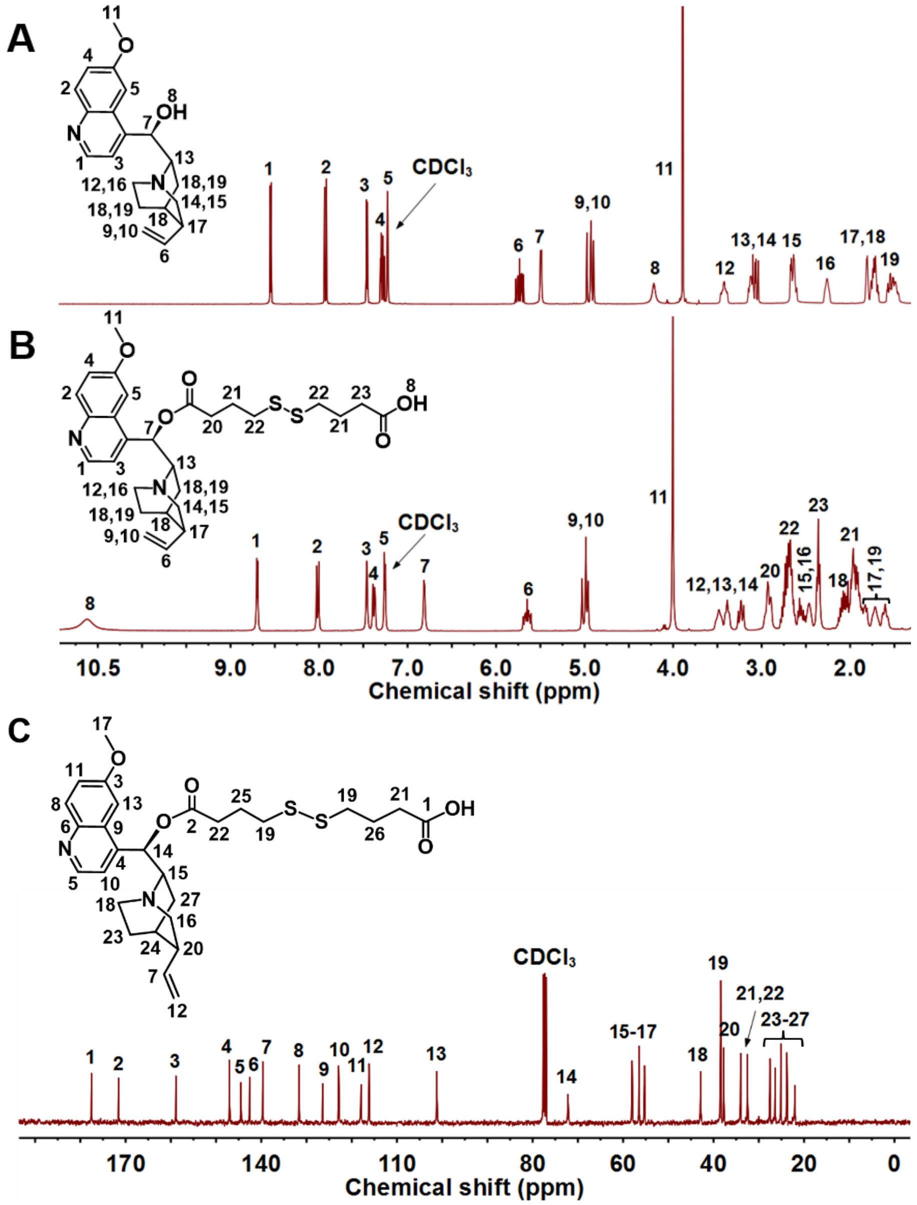

Figure S1 ^{1}H NMR spectra of Qu (**A**) and Qu-ss-COOH (**B**) in $CDCl_3$. (**C**) ^{13}C NMR spectrum of Qu-ss-COOH in $CDCl_3$.

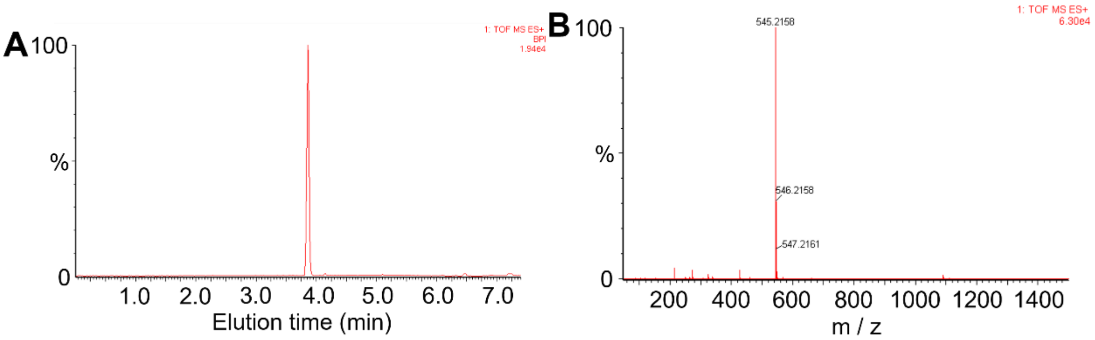

Figure S2 (**A**) LC elution trace, and (**B**) mass spectrum for Qu-ss-COOH.

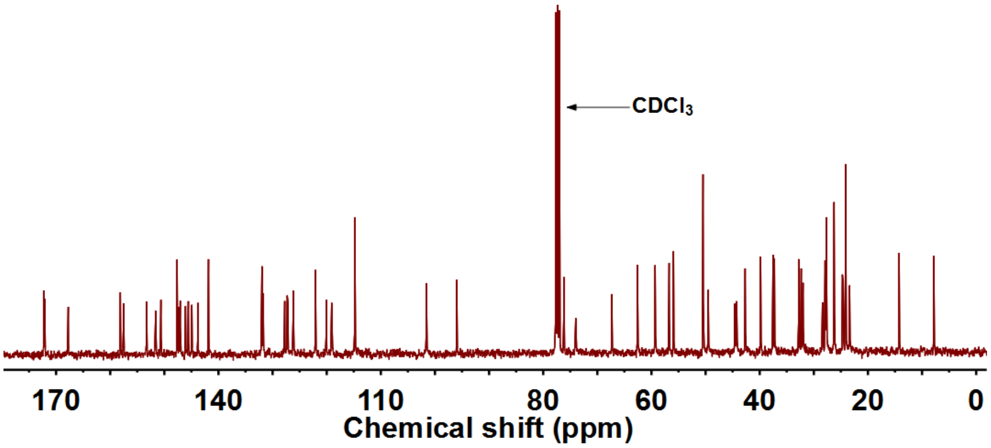

Figure S3 ^{13}C NMR spectrum of Ir-ss-Qu conjugate in $CDCl_3$.

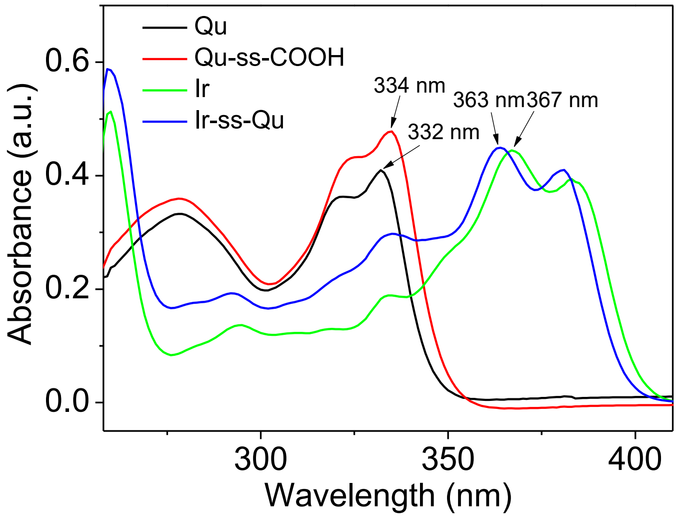

Figure S4 UV/Vis spectra of Qu, Qu-ss-COOH, Ir, and Ir-ss-Qu conjugate in DMSO.

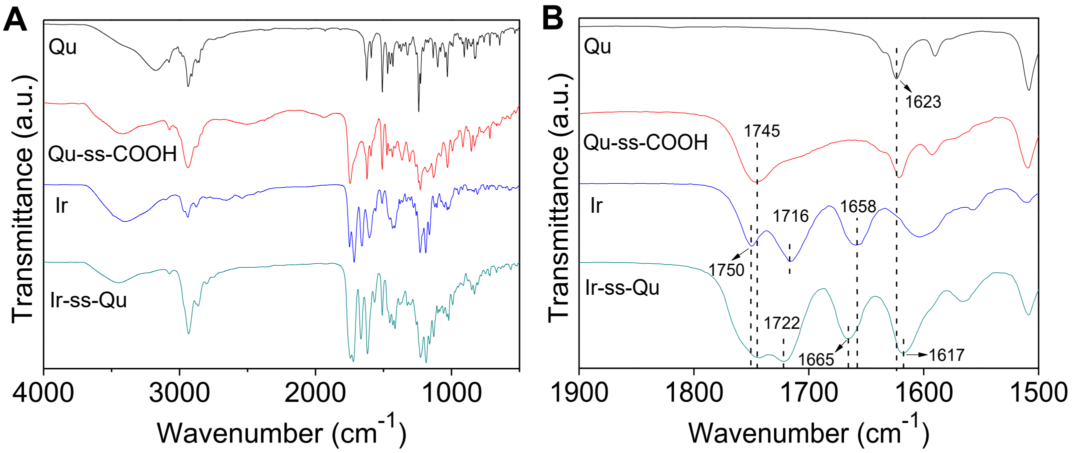

Figure S5 (A) FTIR spectra of Qu, Qu-ss-COOH, Ir, and Ir-ss-Qu conjugate. (B) A partial FTIR spectra of Qu, Qu-ss-COOH, Ir, and Ir-ss-Qu conjugate in the range of 1500 ~ 1900 cm^{-1}.

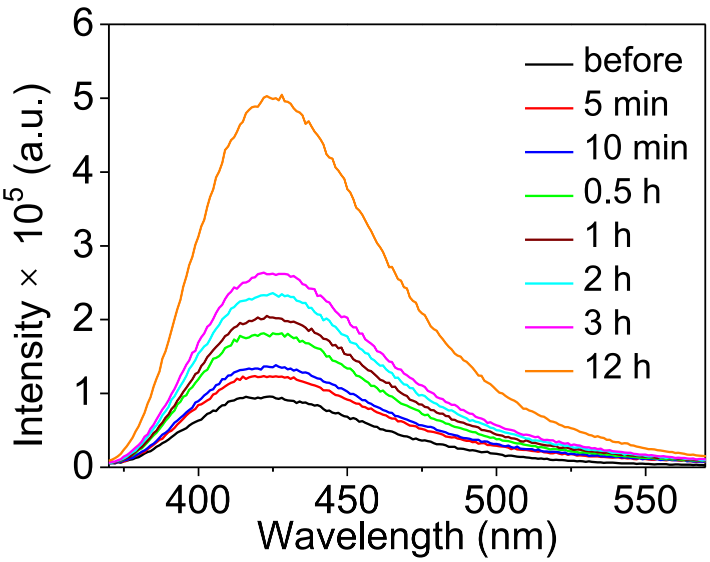

Figure S6 The fluorescence emission spectra (λex = 360 nm, λem = 425 nm) of Ir-ss-Qu conjugate (0.5 μM) in water treated with 5 mM GSH for different time.

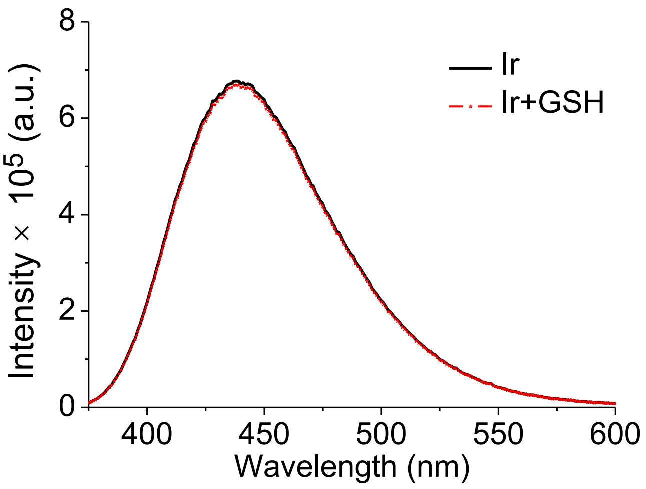

Figure S7 Fluorescence emission spectra (λex = 360 nm, λem = 438 nm) of Ir (0. 5 μM in water) and that after treated by glutathione (GSH, 5 mM) for 12 h.

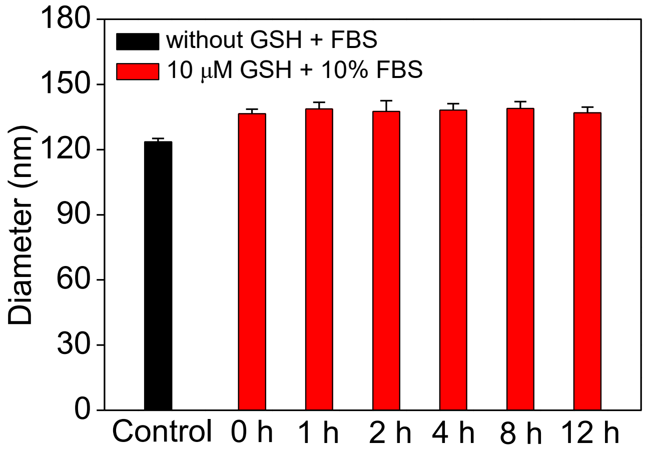

Figure S8 Time-dependent stability of Ir-ss-Qu NPs in water containing 10 μM GSH and 10% FBS. Diameters were measured using DLS. Nanoparticles without GSH and FBS were used as a control.

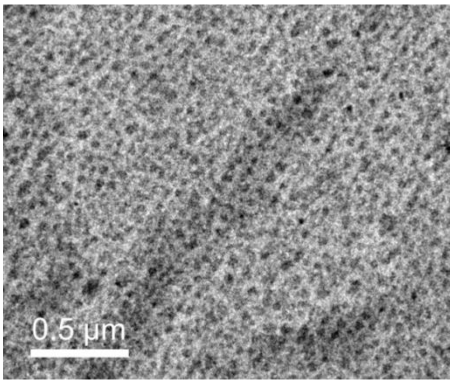

Figure S9 TEM image of Ir-ss-Qu NPs incubated with 1 mM GSH for 0.5 h.

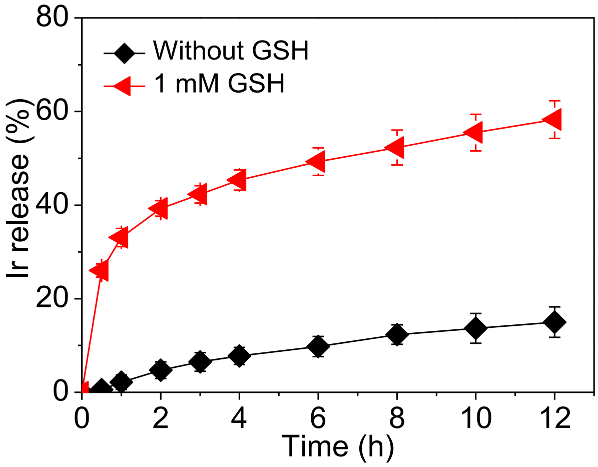

Figure S10 *In vitro* Ir release profiles from Ir-ss-Qu conjugate NPs in PBS with or without 1.0 mM GSH at 37 °C. Data are presented as the average ± standard deviation (n = 3).

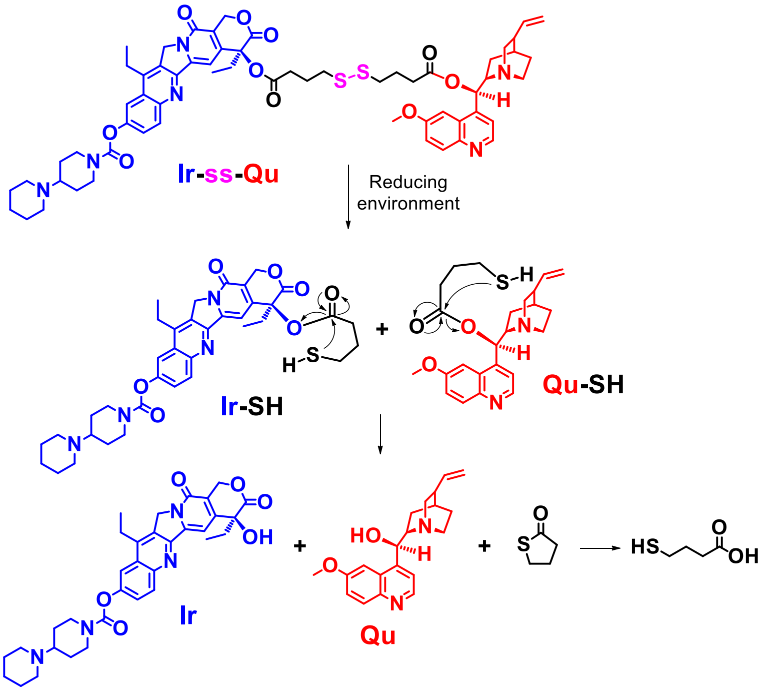

Figure S11 The mechanism of Ir-ss-Qu conjugate reverts to the anticancer drug Ir and inhibitor Qu within the reducing environment.

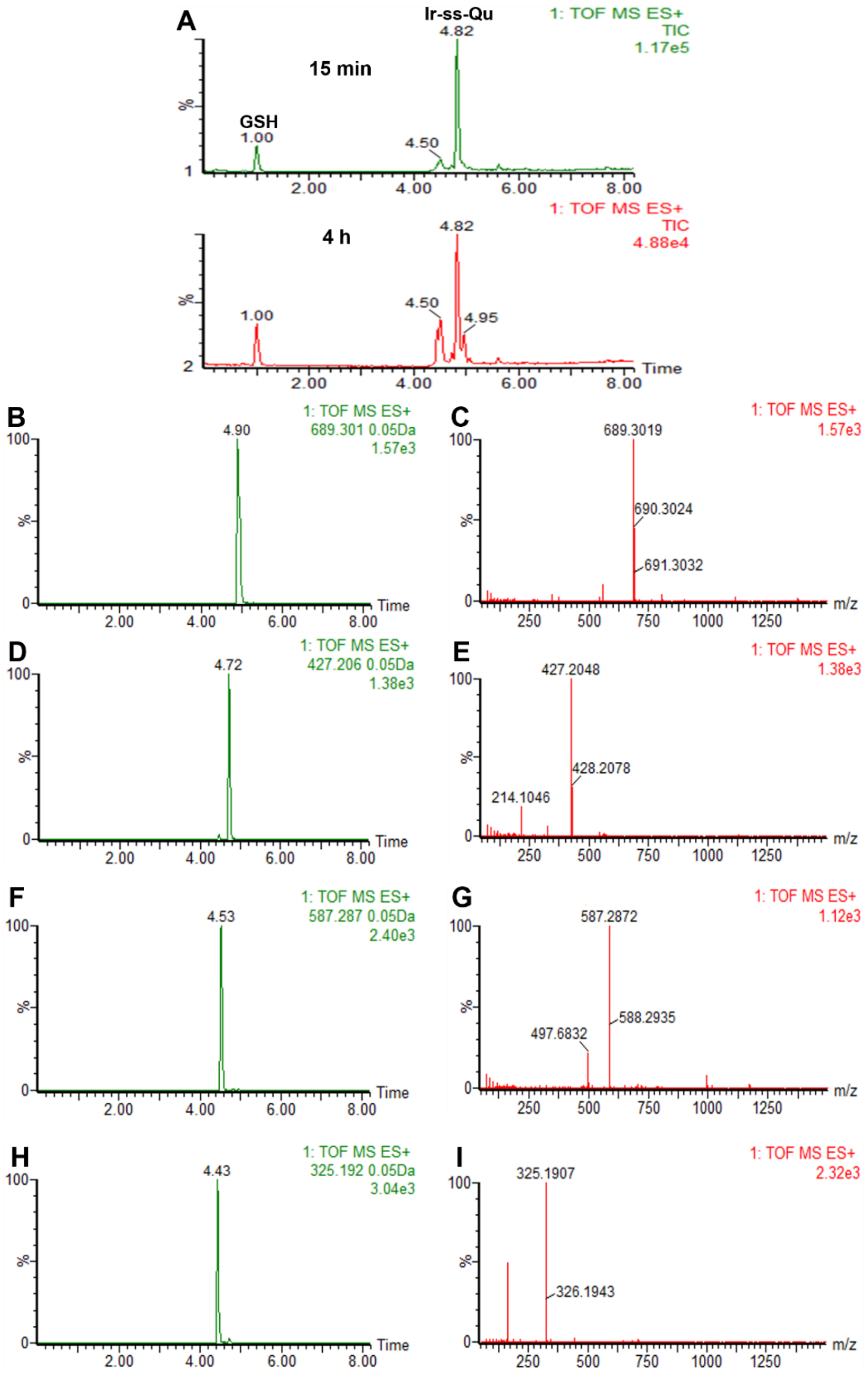

Figure S12 (A) Total ion chromatography (TIC) of Ir-ss-Qu conjugate after treated with GSH for 15 min (top) and 4 h (bottom). (B,D,F,H) Extracted ion chromatography (EIC) of Ir-SH (m/z = 689.3009, ($M+H^+$)), Qu-SH (m/z = 427.2055, ($M+H^+$)), Ir (m/z = 587.2870, ($M+H^+$)) and Qu (m/z = 325.1916, ($M+H^+$)), respectively. (C,E,G,I) The retention time of Ir-SH ($M+H^+$, m/z = 689.3019), Qu-SH (m/z = 427.2048, ($M+H^+$)),

Ir (m/z = 587.2872, (M+H$^+$)) and Qu (m/z = 325.1907, (M+H$^+$)), is 4.90, 4.72, 4.53 and 4.43 min, respectively.

In order to confirm whether the Ir-ss-Qu conjuagte is converted into free Ir and Qu resulting from disulfide bond cleavage under reductive environment, the Ir-ss-Qu conjugate after treated with GSH was characterized by LC-MS technique. As the reducing mechanism of Ir-ss-Qu shown in the Fig. S11, the Ir-ss-Qu firstly was converted to Ir-SH and Qu-SH, subsequently to free Ir and Qu. Fig. S12 gives the molecular weight and retention time of products after disulfide cleaved with GSH-trigged, such as, Ir-SH (M+H$^+$, m/z = 689.3019, at 4.90 min), Qu-SH (m/z = 427.2048, (M+H$^+$), at 4.72 min), Ir (m/z = 587.2872, (M+H$^+$), 4.53 min) and Qu (m/z = 325.1907, (M+H$^+$), at 4.43 min), respectively. The LC-MS results verify that Ir and Qu are obtained eventually after treatment of Ir-ss-Qu conjugate with GSH followed the mechanism.

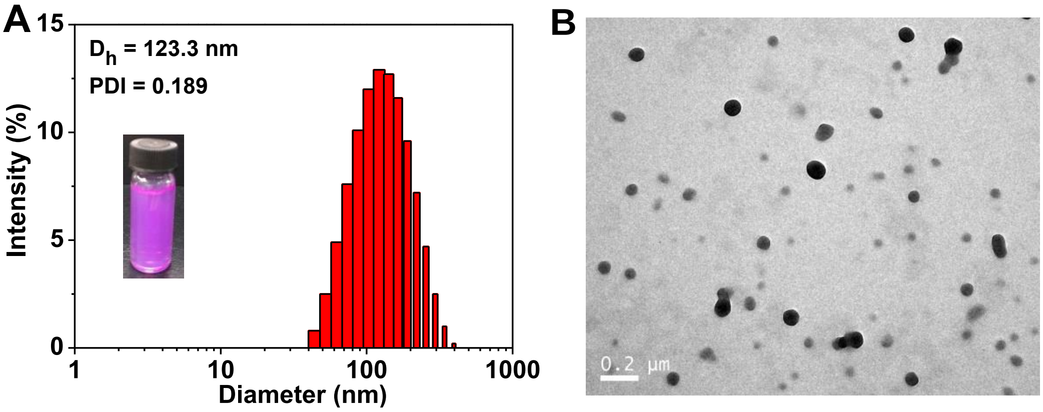

Figure S13 (**A**) DLS measurement of NR-loaded Ir-ss-Qu conjugate NPs. (**B**) The TEM image of NR-loaded Ir-ss-Qu conjugate nanoparticles (scale bar is 0.2 μm).

Table S1. The IC_{50} value and index of drug resistance (IDR)[a] of Ir against MCF-7 and MCF-7/ADR cells.

Cells	IC_{50} (μM)	IDR
MCF-7	7.3	-
MCF-7/ADR	178.6	24.47

[a]IDR = $IC_{50\ (MCF\text{-}7/ADR)}$/ $IC_{50\ (MCF\text{-}7)}$

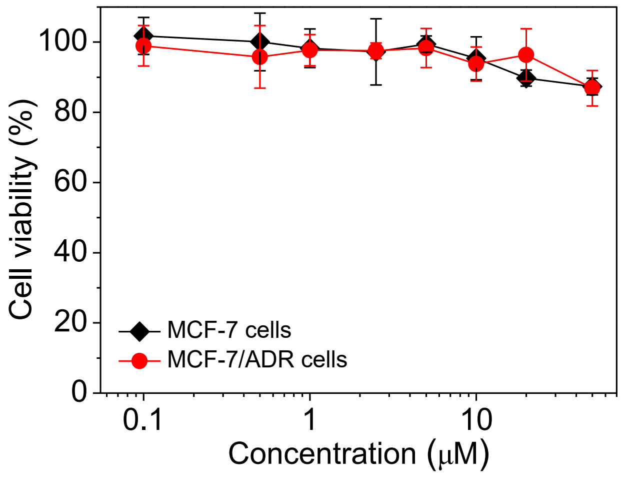

Figure S14 *In vitro* cytotoxicity of Qu to MCF-7 cells and MCF-7/ADR cells determined by MTT assay after 72 h incubation. The data are presented as average ± standard error (n = 6).

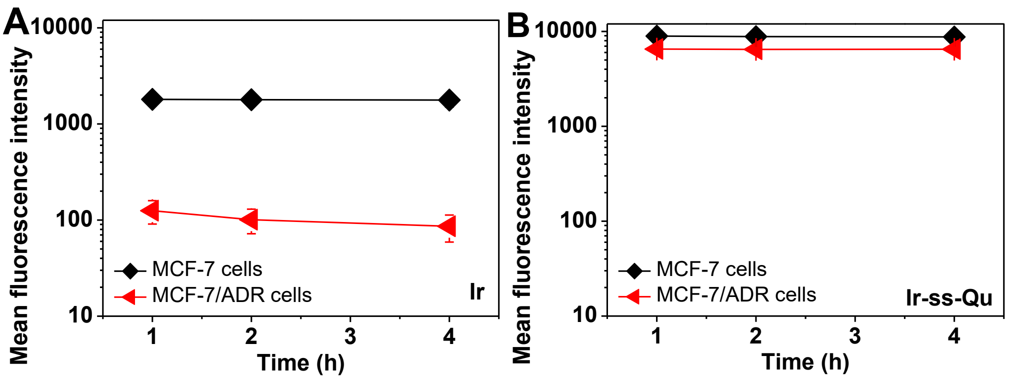

Figure S15 (**A**) The efflux of free Ir from MCF-7 cells and MCF-7/ADR cells. (**B**) The efflux of Ir-ss-Qu conjugate NPs from MCF-7 cells and MCF-7/ADR cells. The cells were first treated with free Ir and Ir-ss-Qu conjugate NPs for 4 h, and then incubated with fresh medium for various time.

In MCF-7 cells, high intracellular drug concentration is observed for free Ir, whereas the amount of free Ir is extremely low in MCF-7/ADR cells and decreases with incubation time as a result of efflux effect of P-gp (Figure S15A). However, the situation is quite different for Ir-ss-Qu conjugate nanoparticles: the drug content is significantly high in both MCF-7 cells and MCF-7/ADR cells. Especially, the concentration of Ir-ss-Qu conjugate still maintains at a high level in MCF-7/ADR cells even after incubation for 4 h (Figure S15B).

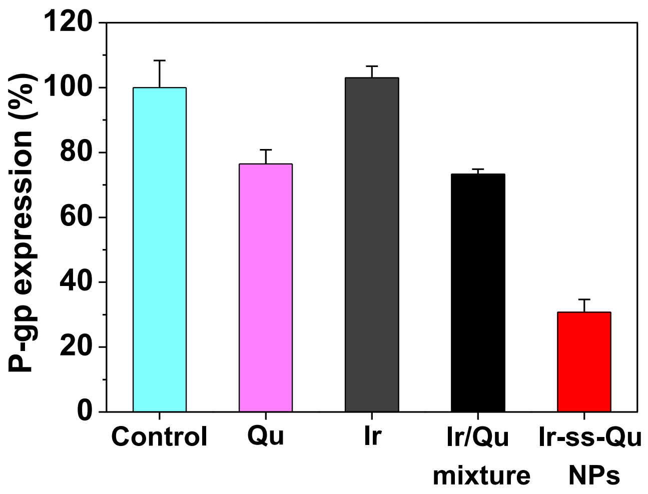

Figure S16 P-gp expression in MCF-7/ADR cells treated with Qu, Ir, Ir/Qu mxiture, and Ir-ss-Qu conjugate NPs. MCF-7/ADR cells untreated are used as a control.

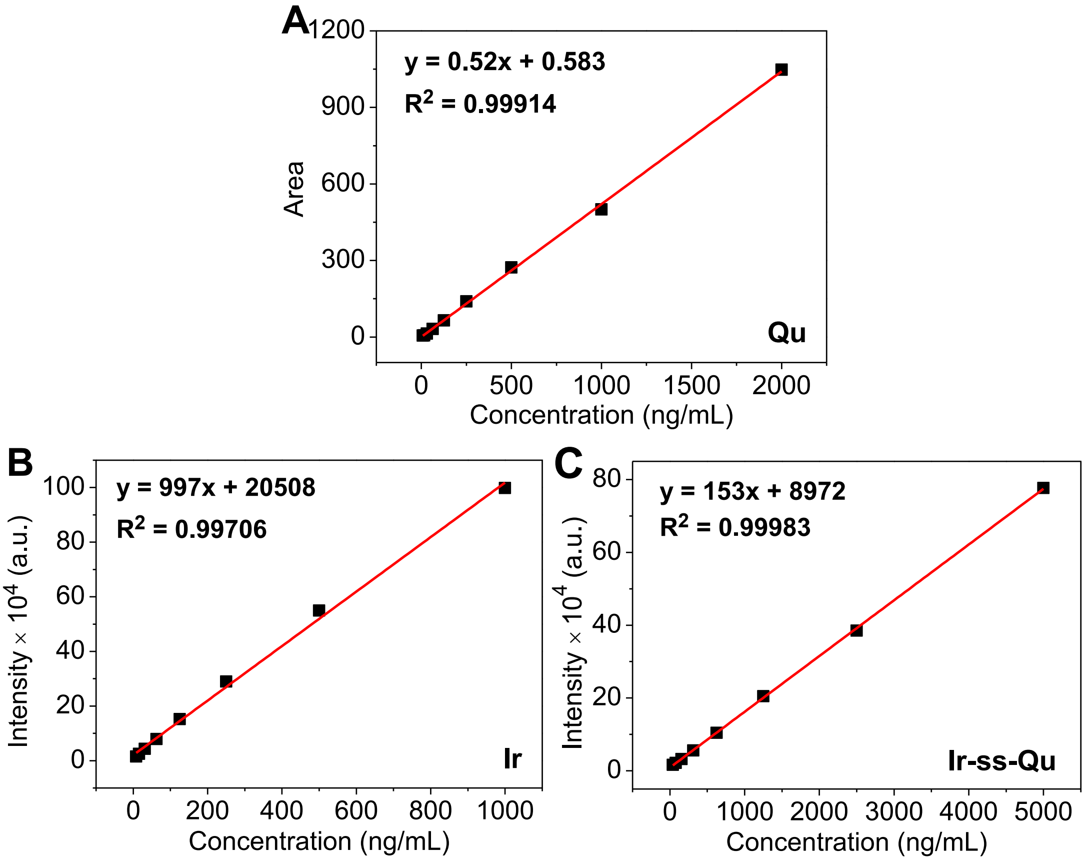

Figure S17 The calibration curves of Qu (A), Ir (B) and Ir-ss-Qu (C) for the PK and BD studies.

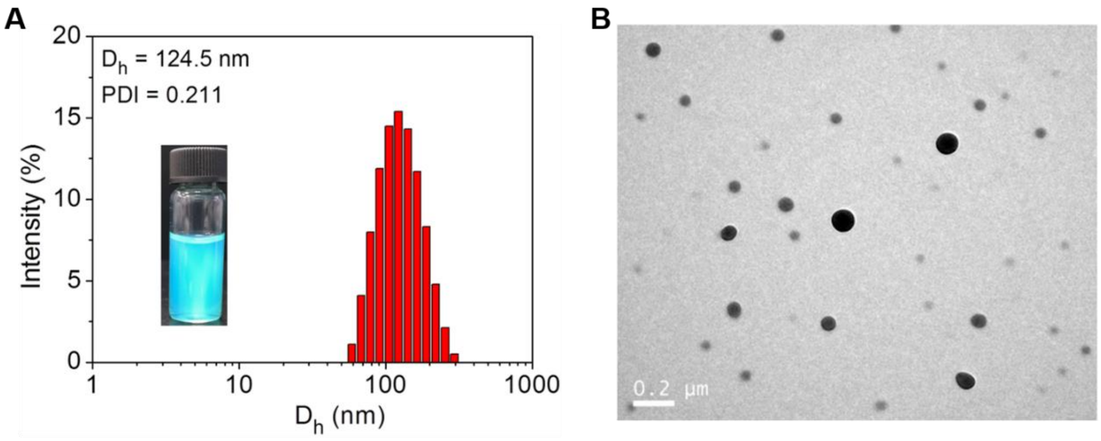

Figure S18 (**A**) DLS measurement of Cy5.5-loaded Ir-ss-Qu conjugate NPs, (**B**) The TEM image of Cy5.5-loaded Ir-ss-Qu conjugate NPs (scale bar: 0.2 μm).

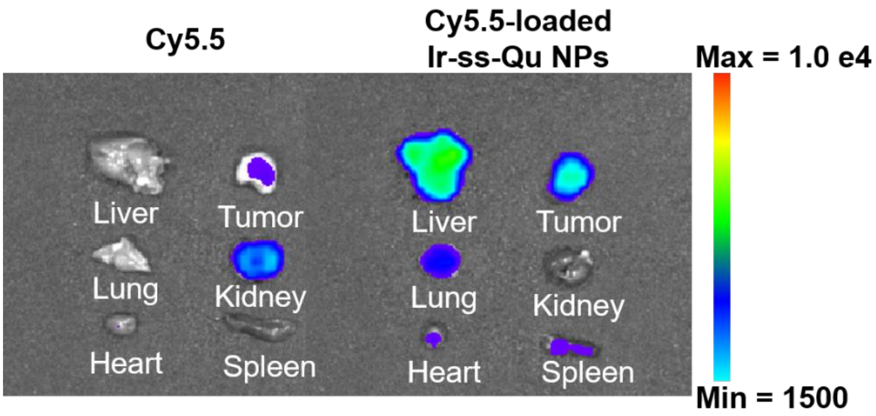

Figure S19 Ex vivo fluorescence images of tumor and organs isolated from MCF-7/ADR tumor-bearing nude mice after intravenous injection of free Cy5.5 and Cy5.5-loaded Ir-ss-Qu conjugate NPs for 24 h.

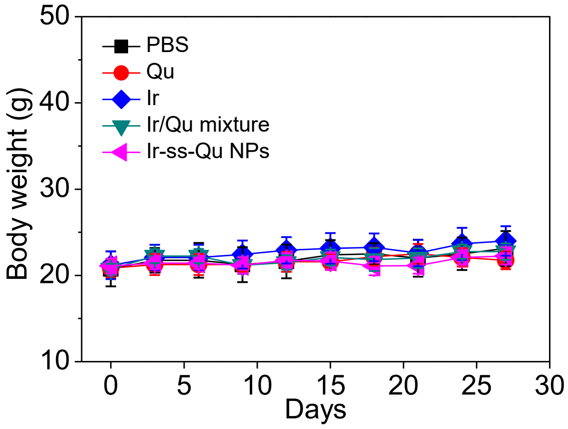

Figure S20 Body weight changes of MCF-7/ADR tumor-bearing mice after treatment with PBS, Qu, Ir, Ir/Qu mixture and Ir-ss-Qu conjugate NPs.

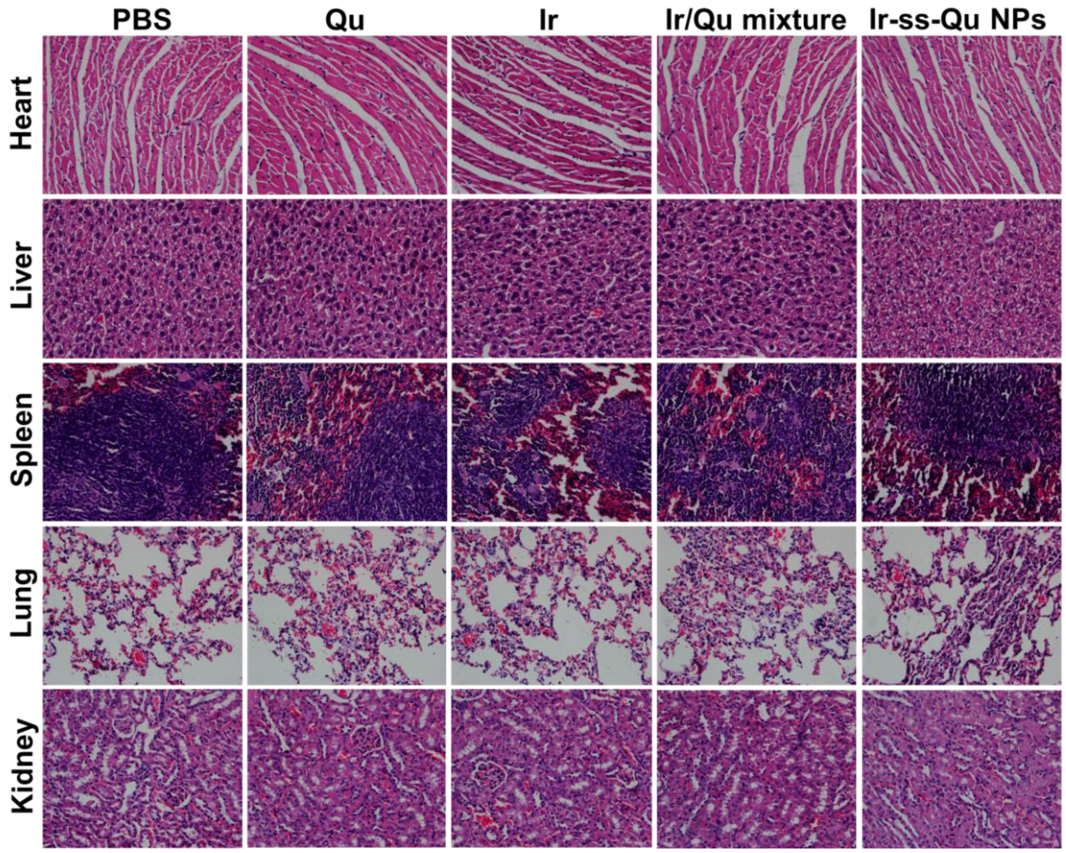

Figure S21 The H&E photographs of heart, liver, spleen, lung and kidney sections of MCF-7/ADR tumor-bearing mice after being treated by intravenous injection of PBS (**A**), Qu (**B**), Ir (**C**), Ir/Qu mixture (**D**), and Ir-ss-Qu conjugate NPs (**E**) for 27 days. (magnification × 400).
